# Same-day genomic and epigenomic diagnosis of brain tumors using real-time nanopore sequencing

**DOI:** 10.1007/s00401-017-1743-5

**Published:** 2017-06-21

**Authors:** Philipp Euskirchen, Franck Bielle, Karim Labreche, Wigard P. Kloosterman, Shai Rosenberg, Mailys Daniau, Charlotte Schmitt, Julien Masliah-Planchon, Franck Bourdeaut, Caroline Dehais, Yannick Marie, Jean-Yves Delattre, Ahmed Idbaih

**Affiliations:** 1 Inserm U 1127, CNRS UMR 7225, Sorbonne Universités, UPMC Univ Paris 06 UMR S 1127, Institut du Cerveau et de la Moelle épinière (ICM), Paris, France; 2 Department of Neurology, Charité-Universitätsmedizin Berlin, Berlin, Germany; 3 Berlin Institute of Health (BIH), Berlin, Germany; 4 Service de Neuropathologie, AP-HP, Hôpitaux Universitaires La Pitié Salpêtrière-Charles Foix, Paris, France; 5 OncoNeuroTek, Paris, France; 6 Division of Genetics and Epidemiology, The Institute of Cancer Research, Sutton, Surrey, SM2 5NG UK; 7 Division of Biomedical Genetics, Center for Molecular Medicine, Department of Genetics, University Medical Center Utrecht, Utrecht, The Netherlands; 8 Department of Genetics, Institut Curie, PSL Research University, Paris, France; 9 Service de Neurologie, AP-HP, Hôpitaux Universitaires La Pitié Salpêtrière-Charles Foix, 2-Mazarin, Paris, France; 10 Laboratory of Translational Research in Pediatric Oncology, Institut Curie, PSL Research University, Paris, France

**Keywords:** Nanopore sequencing, Brain tumor, Glioma, Whole genome sequencing, Epigenomics, Molecular neuropathology

## Abstract

Molecular classification of cancer has entered clinical routine to inform diagnosis, prognosis, and treatment decisions. At the same time, new tumor entities have been identified that cannot be defined histologically. For central nervous system tumors, the current World Health Organization classification explicitly demands molecular testing, e.g., for 1p/19q-codeletion or IDH mutations, to make an integrated histomolecular diagnosis. However, a plethora of sophisticated technologies is currently needed to assess different genomic and epigenomic alterations and turnaround times are in the range of weeks, which makes standardized and widespread implementation difficult and hinders timely decision making. Here, we explored the potential of a pocket-size nanopore sequencing device for multimodal and rapid molecular diagnostics of cancer. Low-pass whole genome sequencing was used to simultaneously generate copy number (CN) and methylation profiles from native tumor DNA in the same sequencing run. Single nucleotide variants in *IDH1*, *IDH2*, *TP53*, *H3F3A,* and the *TERT* promoter region were identified using deep amplicon sequencing. Nanopore sequencing yielded ~0.1X genome coverage within 6 h and resulting CN and epigenetic profiles correlated well with matched microarray data. Diagnostically relevant alterations, such as 1p/19q codeletion, and focal amplifications could be recapitulated. Using ad hoc random forests, we could perform supervised pan-cancer classification to distinguish gliomas, medulloblastomas, and brain metastases of different primary sites. Single nucleotide variants in *IDH1*, *IDH2,* and *H3F3A* were identified using deep amplicon sequencing within minutes of sequencing. Detection of *TP53* and *TERT* promoter mutations shows that sequencing of entire genes and GC-rich regions is feasible. Nanopore sequencing allows same-day detection of structural variants, point mutations, and methylation profiling using a single device with negligible capital cost. It outperforms hybridization-based and current sequencing technologies with respect to time to diagnosis and required laboratory equipment and expertise, aiming to make precision medicine possible for every cancer patient, even in resource-restricted settings.

**Electronic supplementary material:**

The online version of this article (doi:10.1007/s00401-017-1743-5) contains supplementary material, which is available to authorized users.

## Introduction

Histomolecular classification of brain tumors has entered clinical routine diagnostics as the current World Health Organization (WHO) classification explicitly demands histological findings to be refined by molecular testing [20]. Thus, pathologists rely on timely and accurate molecular testing to make an integrated diagnosis using both in situ methods and genetic information. However, high turnaround time of current implementations delays integrated diagnosis by weeks. In addition, targeted next-generation sequencing panels, microarray-based analysis of copy number (CN), and epigenetic alterations all provide high-quality data and aid in the diagnosis and therapeutic management of patients (i.e., stratification or identification of actionable targets or inclusion in clinical trials), but their high capital cost, demanding workflows and need for highly skilled personnel hinder their widespread use. Here, we demonstrate that real-time molecular genomics using nanopore sequencing is both fast and reliable to aid diagnosing cancer by unsupervised classification of CN and methylation profiles.

Nanopore sequencing interprets changes in ionic currents observed when single DNA molecules pass through a nanometer-size protein pore. This has led to the development of handheld size devices that allow sequencing outside of classical laboratory settings and even in the field [27]. While overall throughput currently lacks behind other deep sequencing technologies, nanopores allow read analysis in real-time and selective sequencing [19], both of which allow rapid generation of data. In addition, nanopores are able to discriminate not only the nucleotides of a strand of DNA but also single base modifications such as 5-methylation of cytosine [29, 35]. This allows concurrent analysis of sequence identity and methylation using native DNA.

## Materials and methods

### Experimental design

We performed a retrospective observational study for molecular characterization of diagnostically relevant genetic alterations using nanopore sequencing. Patients were recruited at the Pitié-Salpêtrière university hospital and have given informed consent for research use of tumor material, including genotyping. All tumor samples have been molecularly characterized previously using short-read exome sequencing, Sanger sequencing, SNP array, and/or genome-wide methylation microarray [14, 30].

### Nanopore whole genome sequencing

DNA quality of fresh-frozen tumor tissue was determined using NanoDrop (Thermo Fisher Scientific) and samples were quantified using a QuantiFluor dsDNA assay (Promega, Madison, WI, USA). For whole genome sequencing, libraries were prepared using Rapid 1D Sequencing Kit (SQK-RAD001, SQK-RAD002, or SQK-RBK001, Oxford Nanopore Technologies, UK) following the manufacturer’s instructions. Briefly, 200 ng of tumor DNA was fragmented using a transposase and subjected to adapter ligation. Sequencing was performed using R9 or R9.4 flow cells on a MinION Mk 1B device (Oxford Nanopore) with the MinKNOW software (versions 1.0.5–1.5.12), respectively. For samples run with R9.4 sequencing chemistry, basecalling was performed using Albacore 1.1.0 (Oxford Nanopore). For R9 chemistries, online EPI2ME basecalling (Metrichor Ltd, Oxford, UK) was performed.

Template reads were exported as FASTA using nanopolish or poretools version 0.6 [18] and aligned to the hg19 human reference genome using BWA MEM 0.7.12 with the “−x ont2d” option [17]. Due to compatibility issues of data generated with R9 chemistries, only samples with R9.4 flow cells were used for copy number analysis and methylation-based classification.

### Copy number analysis

For copy number analysis, the QDNAseq package version 1.8.0 [33] and R/Bioconductor, version 3.3, were used. Reads with a minimum mapping quality of 20 were sorted into 1000 kbp bins. Bins with missing reference sequence were excluded from analysis. To account for region- and technology-specific artifacts, public nanopore WGS data for the NA12878 human reference genome were processed identically and subtracted from the normalized tumor sample bin counts. Circular binary segmentation was performed as implemented in the DNAcopy package requiring an alpha value <0.05 to accept change points. Arm-level copy number calls were made by calculating the segment length weighted mean log ratio per chromosome arm.

### Methylation analysis

To identify 5-methylation of cytosines, we used a recently published algorithm based on a hidden Markov model which has been trained using in vitro methylated *E. coli* DNA [35]. Training models for R9 sequencing chemistries were kindly provided by Jared Simpson. We modified the original implementation of nanopolish 0.6.0 to allow methylation calling from different basecalling groups. For classification, the subset of CpG sites overlapping with sites covered by Illumina 450K BeadChip microarrays was used. Beta values in the training set were dichotomized using a cut-off value of 0.6.

### Structural variant detection

For detection of structural variants in amplified regions, we aligned nanopore FASTQ files from sample 3427T to the human reference genome, build GRCh37, using LAST (version 744) with settings: −*Q* 0. The *last-train* function was used with 1000 nanopore reads (~10 million bases) as input to adapt the alignment scoring parameters (−*p*) for error-prone nanopore reads. LAST alignment files (MAF) were converted to BAM files using the *maf*-*convert* function. BAM files were used as input for NanoSV [36] (available at https://github.com/mroosmalen/nanosv) with default settings.

### Amplicon sequencing

Amplicons were designed to cover one or multiple exons of canonical transcripts of *IDH1*, *IDH2*, *TP53*, *H3F3A*, and the *TERT* promoter region. Oligonucleotide primers (Thermo Fisher Scientific) were then designed using Primer3 with the following non-default parameters (*T*_min_ 59 °C, *T*_opt_ 60 °C, *T*_max_ 61 °C, and maximum mononucleotide repeat length = 3) to yield product sizes of 489–2902 bp (Table S1).

25 ng of genomic DNA was amplified using 0.02 U/µl Q5 polymerase (New England Biolabs, Ipswich, MA, USA), 200 µM dNTPs, 500 nM forward and reverse primers, and Q5 reaction buffer with high GC enhancer in a total reaction volume of 20 µl. Thermal cycling was performed as follows: 98 °C initial denaturation for 2 min, followed by 30 cycles of denaturation at 98 °C for 10 s, annealing at 65 °C for 20 s and extension at 72 °C for 90 s, as well as a final extension at 72 °C for 2 min. Amplicons were analyzed using a Caliper LabChip GX DNA 5K assay (Perkin Elmer, Waltham, MA, USA). PCR products were purified using NucleoFast 96 PCR plates (Macherey–Nagel, Düren, Germany).

For amplicon sequencing, Ligation Sequencing Kit 1D (SQK-LSK108, Oxford) was used following the manufacturer’s protocol. Briefly, 1 µg of pooled amplicon DNA was subjected to end repair and dA-tailing. 250 ng of end-repaired DNA (equivalent to 0.2 pmol of 2 kbp fragments) was then used as input for adapter ligation. For real-time monitoring of sequencing depth, reads were streamed to the BWA aligner using npReader [6] with jHDF5 2.11.0 and coverage was calculated using BEDTools [28]. For variant calling, reads were realigned on the event level and variants called using VarScan 2.4.3 [15]. Variants were annotated using SnpEff version 4.3i [9] and ExAC release 0.3.1 germline variants [16] before filtering for coding or hotspot mutations with a minimum mutant allele frequency >0.2.

### Microarray methylation profiling

Samples for Illumina Infinium BeadChip 450K profiling were prepared as described before [14]. Briefly, 500 ng of DNA was subjected to bisulfite conversion. Hybridization and imaging were performed by IntegraGen (Evry, France). Raw IDAT files were preprocessed using the GenomeStudio software (Illumina, San Diego, CA, USA). Processed methylation data from previously characterized samples [14] were retrieved via ArrayExpress (accession E-MTAB-3903). Beta values were used for all the subsequent analysis steps.

### Statistics

All data analysis was done using R/Bioconductor version 3.3 [13]. Hierarchical clustering was used for arranging probes in the depicted classification training set. Random forest classification as implemented in the R/randomForest package, version 4.6–12, was run with default parameters. Sequence concordance was calculated using the Genome Analysis Toolkit’s Genotype Concordance tool, version 3.7 [21].

### Data and material availability

Raw sequencing data are available via the European Genome–phenome Archive (accession EGAS00001002213). Microarray-based methylome data are available at ArrayExpress (E-MTAB-5797). TCGA data were retrieved from the UCSC Cancer Browser [11] or the TCGA FireBrowse website (http://www.firebrowse.org). Pipelines, scripts, and supplementary data to reproduce all results presented in this work are available at https://gitlab.com/pesk/glioma.nano-seq.

## Results

**Fig. 1:**
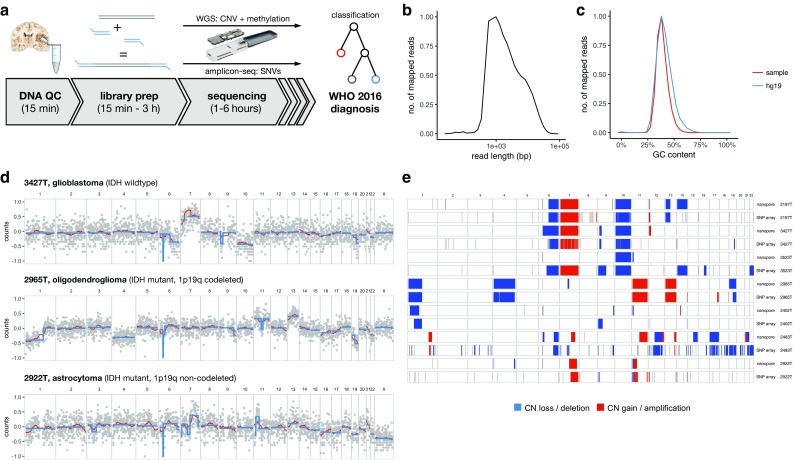
Copy number profiling using nanopore low-pass whole genome sequencing. **a** Same-day workflows to simultaneously characterize copy number variation (CNV) and methylation profiles or single nucleotide variants, respectively. Tumor DNA is subjected to quality control (QC), and then, 250 ng input material is used for library preparation for either whole genome sequencing (WGS) or PCR-based deep amplicon sequencing. **b** Representative read length distribution of mapped reads. Note log scale on *X* axis. **c** Representative distribution of GC content of reads in comparison with the hg19 human reference genome. A randomly drawn subsample of the entire reference genome split into 1000 bp fragments is shown. **d** Copy number profile showing log_2_ transformed, normalized read counts per 1000 kbp window (*grey*) with running mean (*red*) and segmentation results (*blue*). **e** Comparison of nanopore WGS with matched SNP arrays. Heatmaps indicate copy number calls (losses and deletions in *blue*, and gains and amplifications in *red*) across the genome

To meet the needs of the WHO 2016 classification of CNS tumors, we designed 1-day workflows for CN, methylation, and point mutation profiling using nanopore sequencing (Fig. 1a). We first subjected tumor DNA from molecularly well-characterized brain tumors [14, 30] to low-pass whole genome sequencing (WGS) using a commercially available, handheld size nanopore sequencing device. With the aim of widespread implementation in routine diagnostics in mind, we used a transposon-based library preparation kit, which reduces sample preparation time to less than hour. In a cohort of 28 patients (Table 1), low-pass WGS for 6 h performed yielded a mean mapped read depth from <0.01X to 0.24X (Table S1), depending on the sequencing chemistry and input DNA fragment size. Nanopores decipher DNA sequence of single molecules as they present to the pore, generating long reads of variable length, whose distribution is determined by DNA extraction and fragmentation method. We observed typical mean read lengths around 2 kb (Fig. 1b). As library preparation does not involve PCR amplification, no GC bias is introduced and the GC content distribution of the reads resembles closely that of the human reference genome (Fig. 1c).

**Table 1:** Clinical characteristics of patients in study

ID	Age at diagnosis	Sex	WHO 2016 integrated diagnosis	Nanopore sequencing performed	Nanopore methylation-based classification	Key alterations identified by nanopore sequencing
3523T	70	F	Glioblastoma, IDH-wildtype	WGS, amplicon	Not classifiable	pTERT C228T
2197T	58	F	Glioblastoma, IDH-wildtype	WGS, amplicon	Glioma, IDH-wildtype	TP53 p.S241F, pTERT C228T
3427T	72	F	Glioblastoma, IDH-wildtype	WGS, amplicon	Glioma, IDH-wildtype	pTERT C228T, CDKN2A^loss^, EGFR^amp^
2402T	58	M	Anaplastic oligodendroglioma, IDH-mutant, and 1p/19q-codeleted	WGS, amplicon	Not classifiable	IDH1 p.R132H, 1p/19q codeletion, pTERT C228T
2965T	29	F	Anaplastic oligodendroglioma, IDH-mutant and 1p/19q-codeleted	WGS, amplicon	Glioma, IDH-mutant	IDH1 p.R132H, 1p/19q codeletion, pTERT C228T
2483T	51	F	Anaplastic astrocytoma, IDH-mutant	WGS, amplicon	Glioma, IDH-mutant	IDH1 p.R132C TP53 p.R273C, p.R282Q
2922T	44	M	Diffuse astrocytoma, IDH-mutant	WGS	Glioma, IDH-mutant	N/D
6228T	33	F	Diffuse midline glioma, H3.3 K27M-mutant	WGS, amplicon	Classifiable	PDGFRA^amp^
5337T	21	M	Glioma H3.3 G34R	WGS, amplicon	Glioma IDH-wildtype	H3F3A G34R, CDK4^amp^, PDGFRA^amp^
8347T	28	M	Desmoplastic/nodular medulloblastoma, SHH-activated and TP53 wild type	Amplicon	N/D	pTERT C228T
8372T	25	M	Classic medulloblastoma, non-WNT/non-SHH	WGS, amplicon	Medulloblastoma, group 4	pTERT C228T
MB683	7	F	Classic medulloblastoma, WNT-activated	WGS, amplicon	Medulloblastoma, WNT-activated	chr6 loss
8137T	48	M	Anaplastic oligodendroglioma, IDH-mutant and 1p/19q-codeleted	WGS, amplicon	Glioma, IDH-mutant	IDH2 p.R172 W, 1p/19q codeletion, pTERT C228T
8146T	N/A	F	Anaplastic oligodendroglioma, IDH-mutant and 1p/19q-codeleted	WGS, amplicon	Glioma, IDH-mutant	pTERT C228T
7382T	76	F	Glioblastoma, IDH-wildtype	WGS, amplicon	Glioma, IDH-wildtype	pTERT C228T, PDGFRA^amp^ TP53 p.V197M
7455T	45	M	Glioblastoma, IDH-wildtype	WGS, amplicon	Glioma, IDH-wildtype	pTERT C228T
8355T	56	M	Glioblastoma, IDH-wildtype	WGS	Not classifiable	N/D
8356T	73	F	Breast adenocarcinoma, GFAP+, S100+	WGS	Breast cancer	N/D
8357T	79	M	Neuro-endrocrine (prostate adeno) carcinoma, TTF1+	WGS	Lung cancer	N/D
8358T	63	F	Lung adenocarcinoma	WGS	Lung cancer	N/D
8359T	51	M	Bladder urothelial carcinoma	WGS, amplicon	Not classifiable	TP53 p.R280 K
8360T	65	F	Lung adenocarcinoma	Amplicon	N/D	TP53 p.I195T
4596T FFPE	44	F	Anaplastic oligodendroglioma, IDH-mutant and 1p/19q-codeleted	WGS, amplicon	Not classifiable	pTERT C228T
5539T FFPE	28	M	Anaplastic astrocytoma, IDH-mutant	Amplicon	N/D	pTERT C228T^¶^
3718T	78	F	Glioblastoma, IDH-wildtype	WGS	N/D	N/D
3719T	74	M	Glioblastoma, IDH-wildtype	WGS	N/D	N/D
2211T	75	F	Glioblastoma, IDH-wildtype	WGS	N/D	N/D
3724T	65	M	Glioblastoma, IDH-wildtype	WGS	N/D	N/D

Age at initial diagnosis, integrated diagnosis and the type of nanopore sequencing performed are reported. Results of methylation-based random forest classification and key genetic alterations identified by WGS or amplicon sequencing are indicated. Samples were considered not classifiable when there was less than 5 percentage points difference of the majority vote to the next best vote

*WGS* whole genome sequencing, *N/D* not done

^¶^ denotes false-positive variant

### Copy number profiling

We then used WGS data to generate CN profiles. Reads were counted in 1000 kb windows, normalized and subjected to circular binary segmentation (Fig. 1c). No correction of GC bias or mappability is necessary for nanopore reads; however, the long reads cause alignment artifacts with current reference genomes in regions with repetitive sequence such as centromeres. Still, the resulting CN profiles closely resembled matched SNP array-based profiles (Fig. 1d). Importantly, codeletion of chromosome 1p/19q as a diagnostic criterion for oligodendrogliomas implemented in the 2016 WHO classification of CNS tumors was detected in three out of four affected samples (Fig. S1). The remaining sample did not yield sufficient read depth (<0.01) due to low input DNA quality (Table S1). High-level focal amplifications of *EGFR*, *PDGFRA*, and *CDK4* were detected in affected glioblastoma samples (Table 1). In contrast, focal deletions, such as *CDKN2A*, were frequently missed by segmentation. Beyond diagnostic needs, we could reconstruct the double minute nature of an *EGFR* amplification (case 3427T), identify the exact genomic breakpoint using algorithmic structural variant discovery [36], and confirm the latter by Sanger sequencing (Fig. S2).

### Methylation profiling

A major advantage of nanopore sequencing is the ability to detect base modifications, especially 5-methylation of cytosines, in native DNA without need for bisulfite conversion. Epigenomic changes are functionally important in cancer, but also aid in delineating cancer entities. For example, IDH mutations cause a global hypermethylation of CpG islands [25], a phenotype of utmost prognostic importance in neuro-oncology. We thus aimed to detect the G-CIMP phenotype from nanopore reads.

**Fig. 2:**
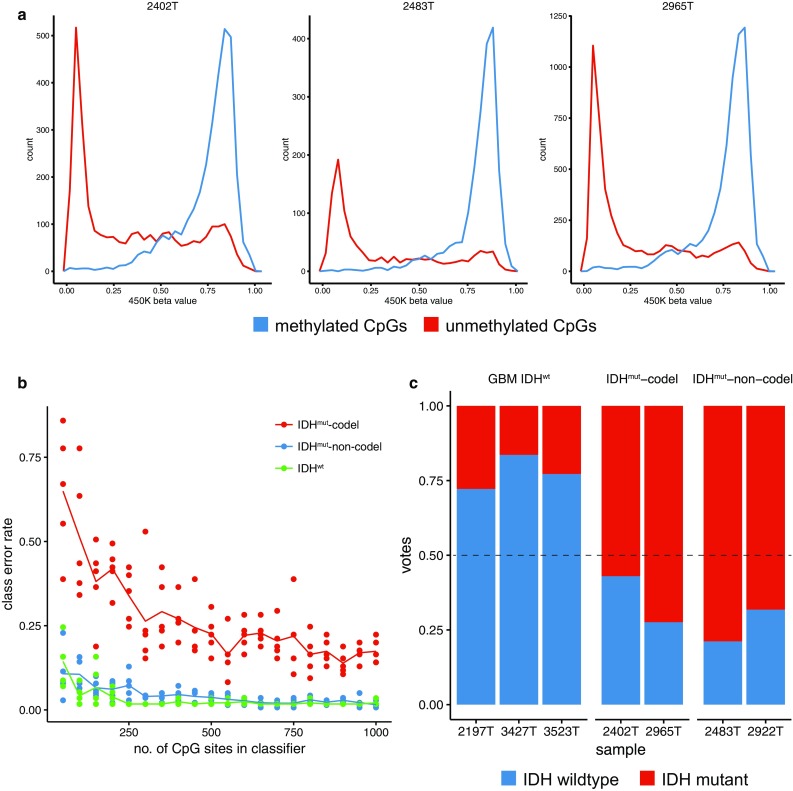
Methylome profiling by nanopore sequencing of native tumor DNA. **a** Comparison of methylation calls from nanopore sequencing with matched Illumina 450K microarray-based data. Beta value distributions for CpG sites that were identified as unmethylated (*red*) or methylated (*blue*), respectively, by nanopore WGS are shown. **b** “Random taiga” simulation of classification error as a function of the number of randomly sampled CpG sites. Each *dot* represents the class-specific error rate of an ad hoc generated random forest using a random subset of *N* CpG sites (indicated on *X* axis) from the TCGA lower grade glioma Illumina 450K cohort as training set. *Lines* indicate the mean of five independent simulations. **c** Methylation profiles from nanopore sequencing discriminate IDH-mutant and wild-type tumors. *Bar plots* indicate vote distribution from ad hoc random forest classification. The TCGA low-grade glioma cohort was used as a training set. Illumina 450K-based beta values were dichotomized using >0.6 as threshold

First, we compared methylation events in CpG sites identified by nanopore sequencing to matched methylome microarrays. Good correlation was observed between single read methylation status of a given CpG site and its corresponding beta value in microarray data (Fig. 2a). Next, we applied random forest (RF) classification to predict IDH mutation.

RF classification is a commonly used machine-learning algorithm based on randomly generated (weak) decision trees [3]. Majority votes then integrate decisions from the entire forest to provide robust classification. The challenge with low-pass WGS data is that it is not known beforehand which CpG sites will be sequenced and the classifier can be built upon. Therefore, we generated random forests ad hoc. With increasing numbers of probed CpG sites, we expect the classifier’s error rate to decrease. To test the feasibility of this approach, we simulated multiple random forests for a given number of CpG sites using the low-grade glioma cohort [5] from The Cancer Genome Atlas (TCGA) and determined misclassification rate for this “random taiga” (Fig. 2b). The simulations show that the mean class error rate to predict IDH and 1p/19q status does not improve for more than approximately 500 CpG sites. This amount of data is reliably sampled within 6 h of nanopore sequencing. Thus, information with respect to a cancer’s entity is redundantly encoded in the methylome and this fact can be exploited for classification from sparse, randomly sampled CpG sites.

Using the same training set, we then predicted IDH status in our samples from nanopore-based methylation calls. Due to the low read depth (usually *N* = 1), methylation calls from nanopore WGS were binary. To enable classification using microarray-based training data, beta values were dichotomized as described in previous applications of RF in methylation data [5, 7]. All samples were correctly classified (Fig. 2c).

### Supervised pan-cancer classification

**Fig. 3:**
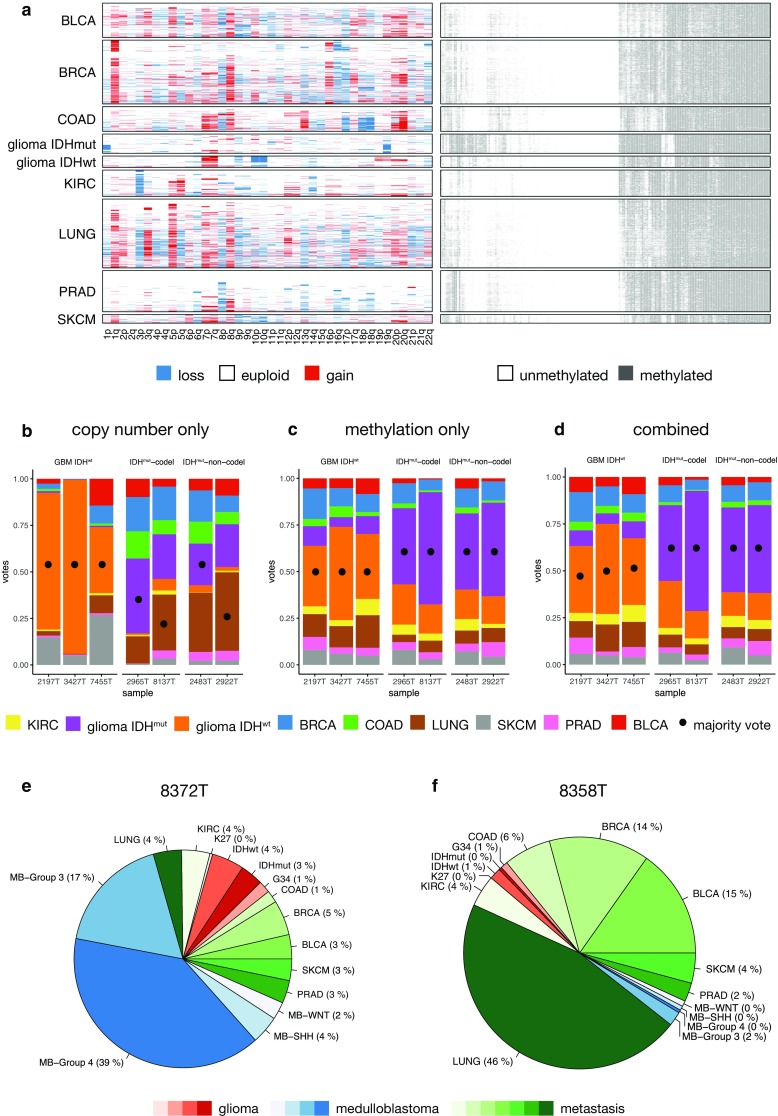
Pan-cancer classification using copy number and methylation profiles. **a** Training set composed of TCGA samples from nine cancer entities using arm-level averaged copy number (CN) information (CN loss *blue*, CN gain *red*) and dichotomized methylation data. For illustration purposes, only 200 random CpG sites were sampled, clustered, and plotted. **b**–**d** Classification of samples subjected to WGS using R9.4 flow cells using ad hoc random forests (500 trees per sample). *Bar plots* show vote distributions based on copy number only (**b**), methylation (**c**), or both modalities (**d**). **e**, **f** Methylation-based pan-cancer classification of medulloblastoma (**e**) and a brain metastasis of a lung adenocarcinoma (**f**). *BRCA* breast cancer, *BLCA* bladder urothelial carcinoma, *COAD* colon adenocarcinoma, *KIRC* kidney renal cell carcinoma, *LUNG* lung squamous cell and adenocarcinoma, *SKCM* skin cutaneous melanoma, *PRAD* prostate adenocarcinoma, *MB* medulloblastoma, *K27* diffuse midline glioma H3 K27M mutant, *G34* pediatric glioblastoma, H3 G34R mutant

Next, following the idea of a machine-learning-based molecular classification of tumors to fully recognize molecular entities and rule out interobserver variability [32], we sought to investigate whether nanopore CN and methylation profiles can be used to classify tumor samples on a pan-cancer level. As a training set for all analyses, we used public microarray-based methylation data from primary brain tumors (adult and pediatric glioblastomas, lower grade gliomas, and medulloblastomas) and tumors that frequently metastasize to the brain (melanoma, breast, lung, bladder, prostate, colon, and clear cell renal carcinoma) [1, 2, 4, 5, 12, 23, 24, 37–40]. Where CN data were available, too, SNP array-based CN profiles were aggregated to chromosome arm level and added to the training set (Fig. 3a). The resulting classifiers for any set of CpG sites in our cohort usually yielded an overall out-of-bag classification error rate ≪5%.

We first subjected seven glioma samples with CN and methylation profiles generated by nanopore sequencing to ad hoc RF classification. When we compared classification using CN alone (Fig. 3b), methylation only (Fig. 3c) or both modalities together (Fig. 3d), using the joint approach improved overall accuracy: all (7/7) samples were correctly classified.

Then, we subjected two medulloblastoma (MB) cases to classification (here, only methylation training data were available). Both samples were identified as MB and also the genetic subtype according to the WHO classification was predicted correctly as WNT-activated (case MB683) or non-SSH-activated/non WNT-activated (i.e., group 4, case 8372T) (Fig. 3e). Next, we attempted classification of brain metastasis and could predict the pulmonary origin in one case (Fig. 3f). We also selected a metastasis of a breast adenocarcinoma in the posterior fossa for study which immunohistochemically showed expression of GFAP and S100, so it was misleading for the diagnosis of carcinoma. Pan-cancer classification based on nanopore WGS correctly identified this sample as breast cancer (Table 1, Fig. S1).

Several cases were not classifiable (requiring a > 5 percentage points’ difference of the majority vote to the next best vote) or misclassified (Table 1). These cases had often lower DNA quality with respect to fragment size (Table S1). One GBM sample that was not classifiable had low tumor purity when estimated from matched transcriptomic profiles using the ESTIMATE algorithm [41] (Fig. S3a). This also resulted in false-negative calling of copy number CN alterations using fixed thresholds, even though they were present at visual inspection (Fig. S3b).

### Amplicon sequencing

**Fig. 4:**
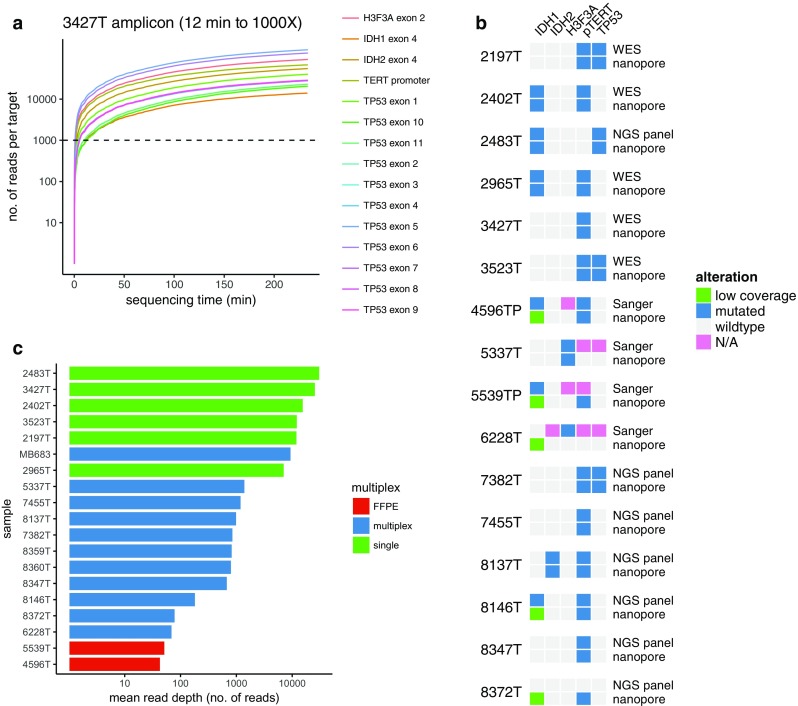
Real-time amplicon sequencing of single nucleotide variants. **a** Representative coverage plot of target regions in *IDH1*, *IDH2*, *H3F3A*, *TP53*, and *TERT* promoter region over time. The time needed to achieve 1000X depth in all amplicons is indicated. Note log scale on *Y* axis. **b** Mean read depth over all amplicons in samples processed individually or as barcoded multiplex libraries. Of note, FFPE samples were sequenced as part of a multiplex library. **c** Comparison of selected variant calls from nanopore sequencing (filtered for coding or hotspot mutations with minimum allele frequency >0.2) with reference calls from Sanger or Illumina sequencing

Finally, we explored deep amplicon nanopore sequencing for identification of single nucleotide variants. We designed an amplicon panel covering hotspot exons in *IDH1*, *IDH2*, and *H3F3A*, all coding exons of *TP53* and, additionally, the *TERT* promoter (pTERT) region. Due to the long reads delivered by nanopore sequencing, this could be achieved with only nine PCR reactions (Table S2). Mutations in these genes (with exception of pTERT) inform molecular diagnosis of glioma and medulloblastoma, and are demanded for diagnosis in the 2016 WHO classification of CNS tumors [20]. Sufficient read depth is a critical parameter for variant calling with defined sensitivity and specificity. We thus implemented a real-time analysis pipeline that allowed monitoring of read depth and to stop sequencing when sufficient information to make a diagnosis has been collected (Fig. 4a). In samples run as single samples with real-time monitoring, a sequencing depth of 1000X in all target regions could repeatedly be achieved within 2–20 min of sequencing. Mean overall coverage >1000X could be achieved in single runs, but was lower in runs using barcoding PCR for multiplexing (Fig. 4b).

In all samples, coding mutations were reliably detected as compared to routine diagnostics based on Sanger sequencing, immunohistochemistry or a next-generation sequencing (NGS) panel (Fig. 4c). Nanopore sequencing reads have historically shown high error rates, especially in homopolymer contexts. We, therefore, compared nanopore consensus sequences to matched short-read whole exome data in five cases. Overall concordance was 97.8–98.6% before functional filtering. Even though at low number (<5 per sample) after filtering for coding mutations, false-positive variants were present. Most of these mutations occurred in multiple samples, indicating a context-specific error (Table S3). Improved base calling algorithms are thus needed to reduce the time to manually review mutations for false positives.

### Technical aspects

Nanopore sequencing is highly scalable due to low capital cost of the device (use of multiple sequencers) and reuse of flow cells. To exclude carry-over and cross-contamination in sequential sequencing runs and for scalability, we evaluated barcoding and multiplexing for both WGS and amplicon workflows (Table S1, Fig. 4b). For WGS, up to four samples were combined without major protocol changes and permitting convenient overnight runs (e.g., one sample for 6 h and two samples for 12 h). Barcoding of amplicon libraries and multiplexing 12 samples greatly reduces per-assay price at the cost of additional PCR and quality control steps. Finally, we explored use of DNA derived from formalin-fixed paraffin-embedded tissue (FFPE). PCR amplicons were generated from two FFPE samples with identical input amount and protocol. As expected from the usually highly fragmented DNA, PCR yields were lower, especially for large amplicons (>1 kbp). This could only partly be compensated by extending sequencing time. For nanopore WGS, transposase-based library preparation is not compatible with fragment size distribution of FFPE-derived DNA samples. We thus performed a different ligation protocol to test WGS in one FFPE sample. While read yield was acceptable (Table S1), the resulting copy number profile was noisy and hard to interpret (Fig. S1). In summary, nanopore sequencing is compatible with FFPE samples, but clearly not recommended due to inferior performance.

## Discussion

Histomolecular classification promises to significantly improve diagnosis, prognosis, and treatment decision making of cancer patients by aiding in clearly delineating distinct (molecular) entities and identifying targetable genomic alterations for personalized treatment. It is, therefore, crucial to ensure widespread implementation of appropriate technology in clinical routine for patient benefit. We explored the potential of nanopore sequencing to comprehensively characterize genetic alterations.

CN alterations could be detected in brain tumor samples using ultra low-pass WGS. While overall resolution is lower than current SNP arrays or NGS approaches, arm-level alterations and high-level focal alterations are reliably recapitulated. Most importantly, detection of 1p/19q-codeletion fulfills diagnostic needs for the current WHO 2016 classification of CNS tumors. While WGS using rapid, transposase-based library preparation works very well with high molecular weight DNA, some of the clinical routine fresh-frozen tumor DNA samples were highly fragmented and yielded insufficient results. Quality of input DNA thus seems to be pivotal. For use of FFPE material, changes to the protocol and further optimization are needed.

Methylation data can directly be obtained from the same WGS data set which makes time-consuming bisulfite conversion and specialized methylation assays (sequencing or hybridization-based) expendable. Very recently, it has been shown in the context of meningioma that classification of tumors using methylome data alone is sufficient or superior to make a correct diagnosis [32]. With low genome coverage, we obtained sparse random sampling of CpG sites. We show that this information is sufficient to subtype gliomas into IDH-mutant vs. wild-type samples and that cancer entities from different tissue origins can be distinguished in a few hours. This may aid in the differential diagnosis of primary brain tumors vs. brain metastases and greatly facilitate staging and the search for unknown primary tumors [22]. However, as diagnosis is inferred from relatively sparse data, it precludes inter-patient comparison and reuse of data with currently obtainable coverage in the (relatively short) time frame of 6 h of sequencing.

Finally, we used PCR-based amplicon generation followed by nanopore sequencing to identify point mutations. Using a small, but diagnostically relevant gene panel (covering target regions with a total of 12 kb), high read depth could be routinely obtained in less than 30 min of sequencing when using real-time depth monitoring. However, context-specific base calling errors introduce platform-specific errors and false variant calls that need to be carefully reviewed.

### Comparison to existing technologies

Targeted next-generation sequencing panels tailored to detect mutations in brain tumors or, more generally, cancer-related genes have been employed routinely with a turnaround time of several days [8, 31]. Methylation-based classification of brain tumors by microarray allows differentiation of a wealth of different entities within 2 weeks [12, 32]. Intraoperative subtyping of gliomas is possible using allele specific PCR for key alterations (IDH1, pTERT) but remains restricted to hotspot point mutations [34]. Similarly, CN changes and mutations have been detected in cell-free DNA from CSF to allow less invasive diagnostics [10, 26]. A major drawback of all approaches is the high investment cost, need for laboratory space or expertise.

For nanopore sequencing, besides the portable sequencing device and a laptop computer, only a spectrometer for DNA quantification and a thermocycler for library preparation and amplicon generation by PCR are needed. This allows implementation of a complete molecular pathology laboratory even in resource-restricted settings or mobile environments. Per sample cost is ~$200 for WGS and ~$120 for amplicon sequencing without multiplexing. However, being a technology still under development, frequent updates in chemistry and software currently challenge routine use and need to be addressed to allow standardized diagnostics across laboratories. In addition, hybridization microarrays and targeted short-read sequencing both work relatively well with fragmented DNA from FFPE samples, while this currently poses a technical challenge for nanopore sequencing.

Our study has several limitations. First, as this is a proof-of-principle study, sample number is small and precludes accurate quantification of sensitivity or specificity to detect structural alterations and point mutations. Second, a prospective and multi-centric evaluation of the approach presented here is needed to rule out sample selection bias and demonstrate robustness across laboratories. Third, we reused flow cells to reduce per-assay cost, but washing also decreased the number of active pores and thus performance in subsequent runs.

In conclusion, same-day diagnosis of CN alterations, epigenetic modifications, and single nucleotide variants using nanopore sequencing is feasible with minimal capital cost and without need for sophisticated laboratory equipment. For CNS tumors, molecular features demanded for diagnosis by current guidelines can be obtained, which, together with histological data and grading, enable accelerated integrated diagnosis and improve patient care.

## Electronic supplementary material

Below is the link to the electronic supplementary material.

Supplementary material 1 (PDF 11720 kb)

Supplementary material 2 (XLSX 38 kb)

Supplementary material 3 (XLSX 9 kb)

Supplementary material 4 (XLSX 28 kb)
