# Supplementary material for: Same-day genomic and epigenomic diagnosis of brain tumors using real-time nanopore sequencing
Source: Acta Neuropathol. 2017 Jun 21;134(5):691–703. doi: 10.1007/s00401-017-1743-5 (PMC5645447; doi:10.1007/s00401-017-1743-5)
Supplement: Supplementary file 1 — Supplementary material 1 (PDF 11720 kb) [file 401_2017_1743_MOESM1_ESM.pdf]

Supplementary Figures S1 – S3

Fig. S1: Sequencing reports for nanopore whole genome sequencing. For each sample that underwent whole genome sequencing, basic quality metrics (read length and GC distribution), the copy number profile and the result of methylation-based pan-cancer random forest classification are shown.

2197T

Quality control metrics

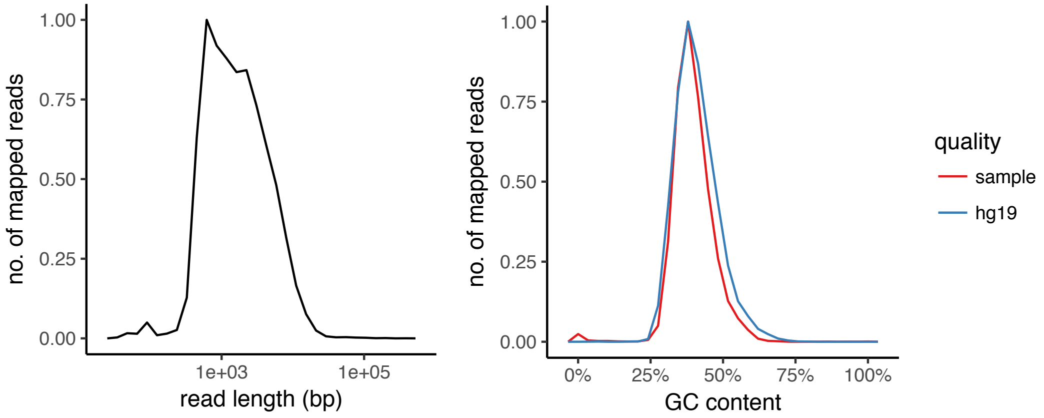

Copy number profile

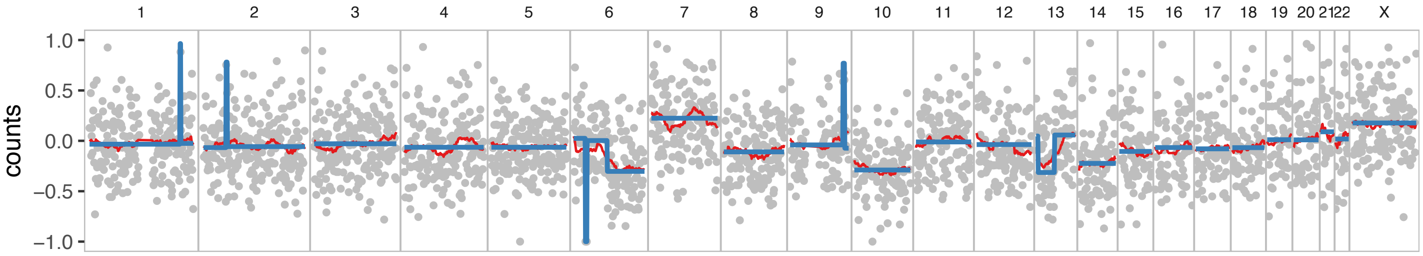

Methylation based classification

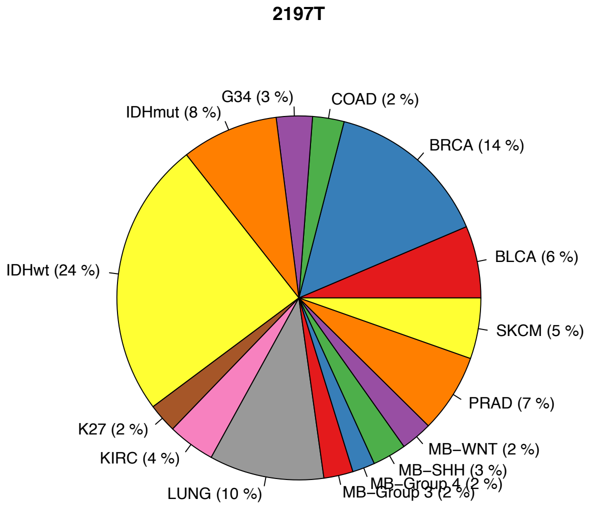

2402T

Quality control metrics

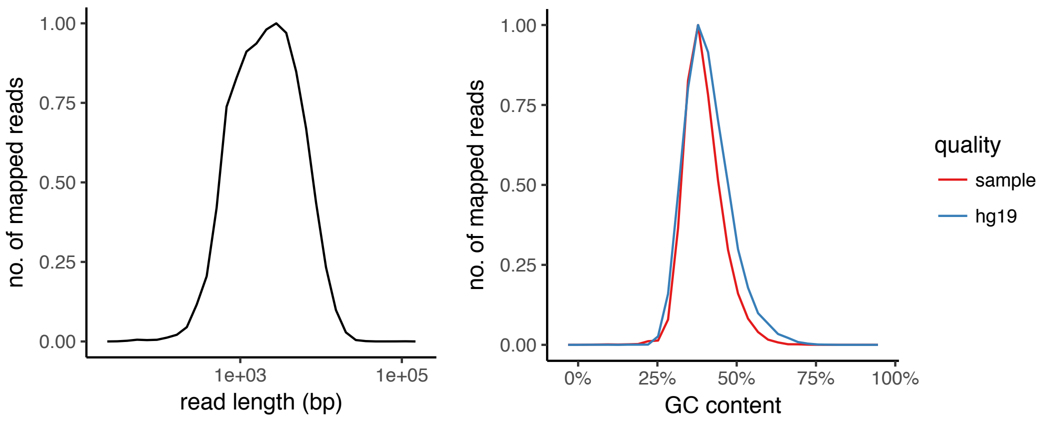

Copy number profile

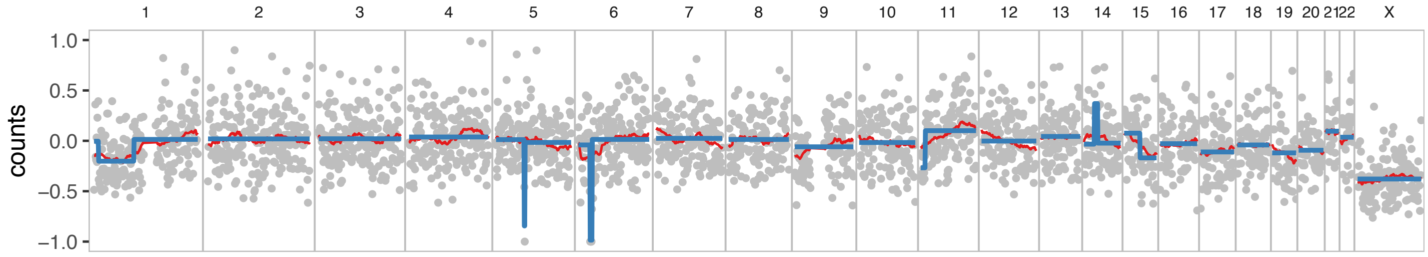

Methylation based classification

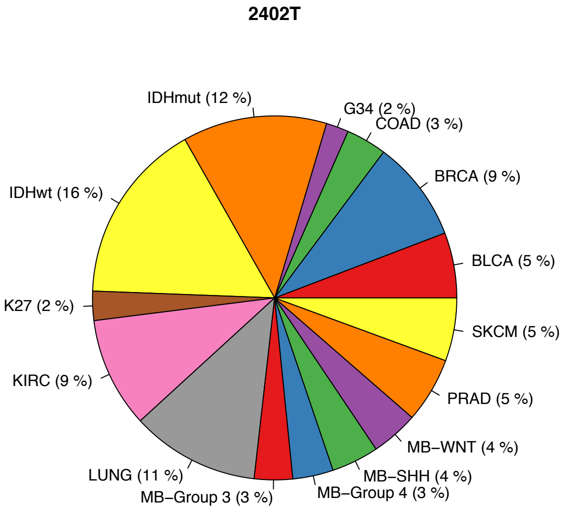

2483T

Quality control metrics

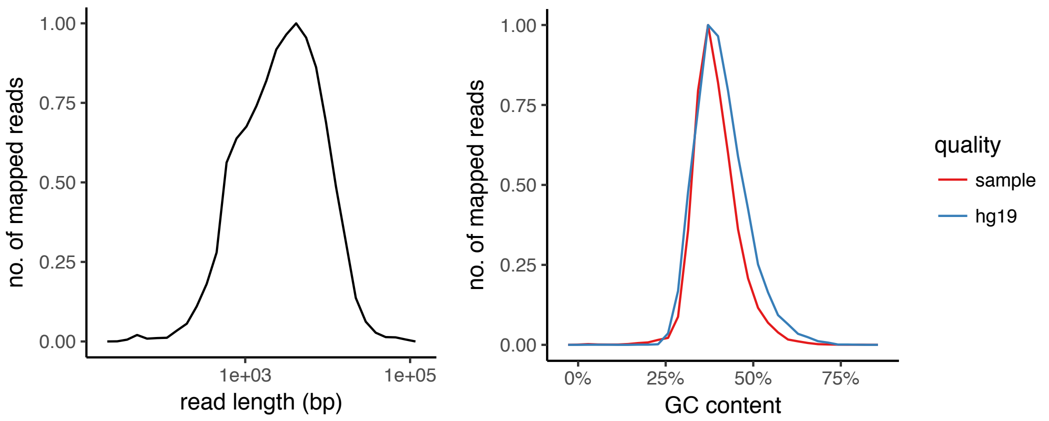

Copy number profile

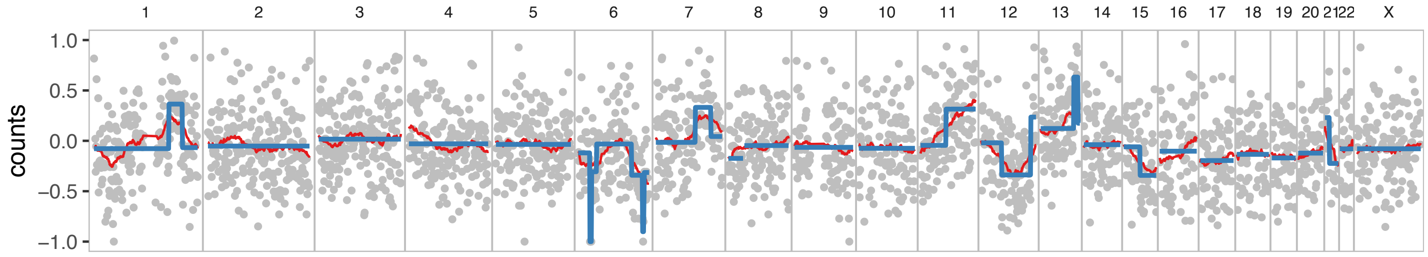

Methylation based classification

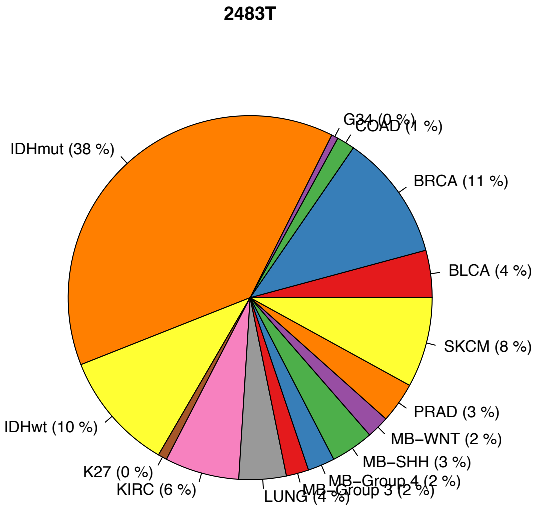

2922T

Quality control metrics

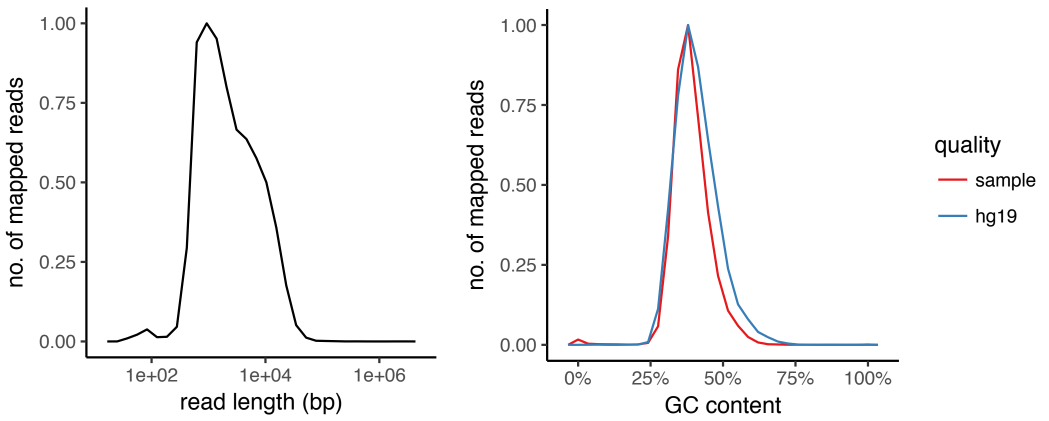

Copy number profile

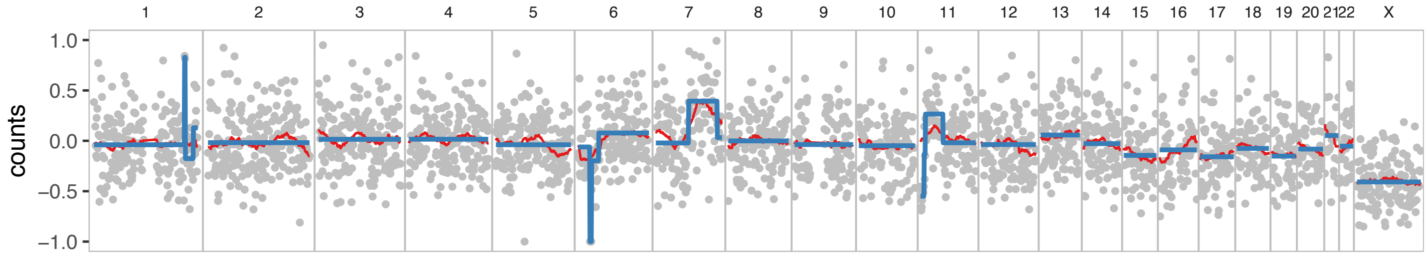

Methylation based classification

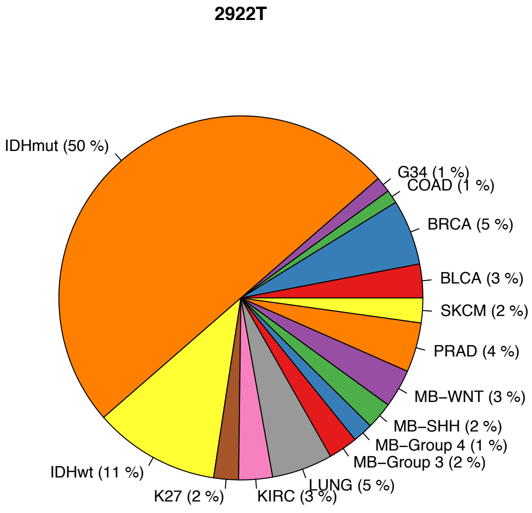

2965T

Quality control metrics

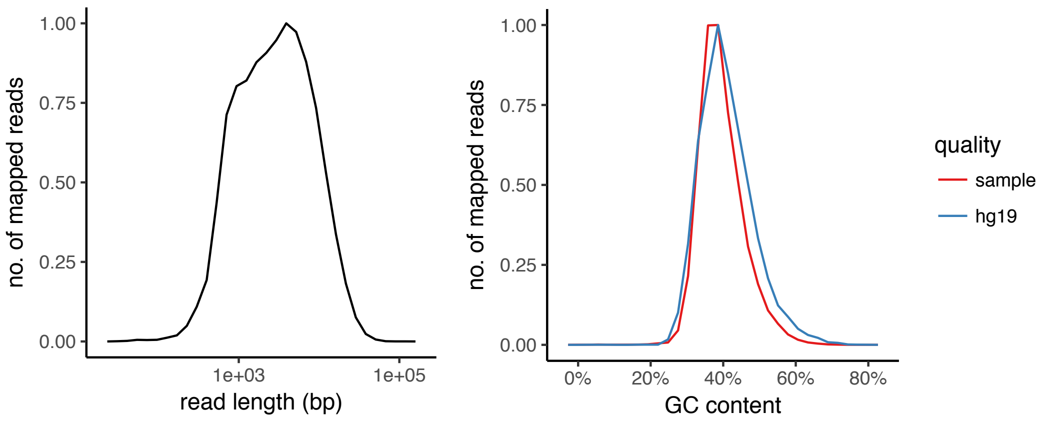

Copy number profile

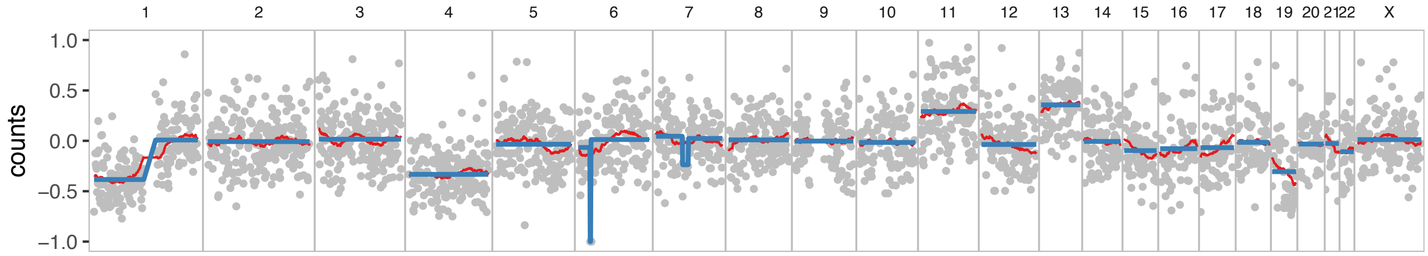

Methylation based classification

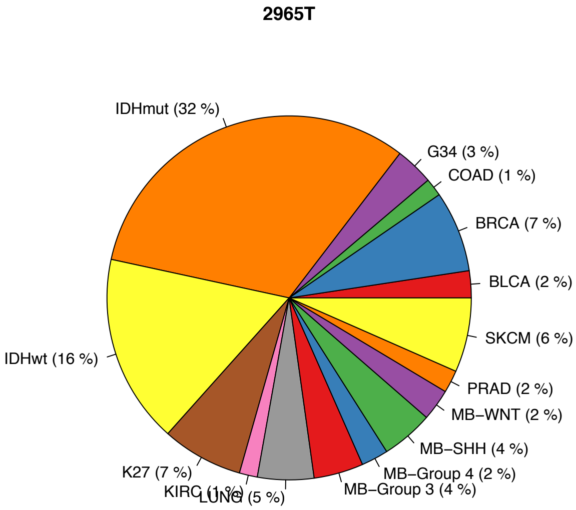

3427T

Quality control metrics

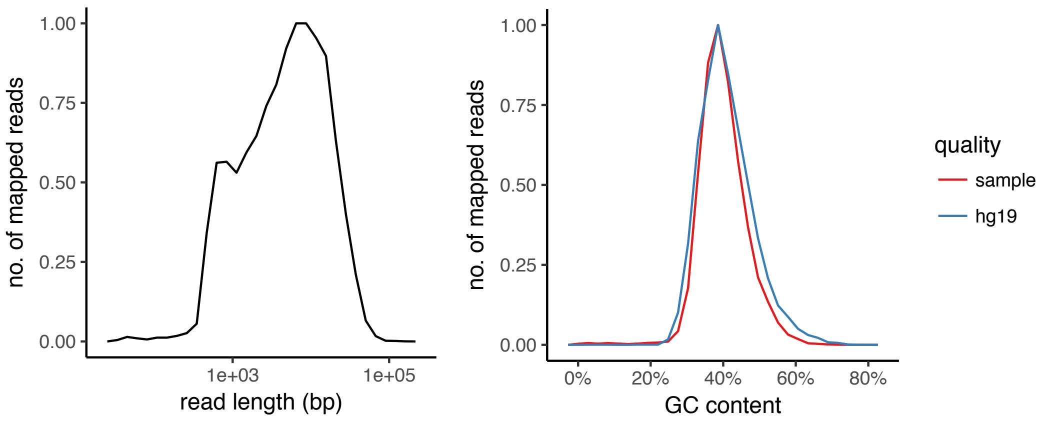

Copy number profile

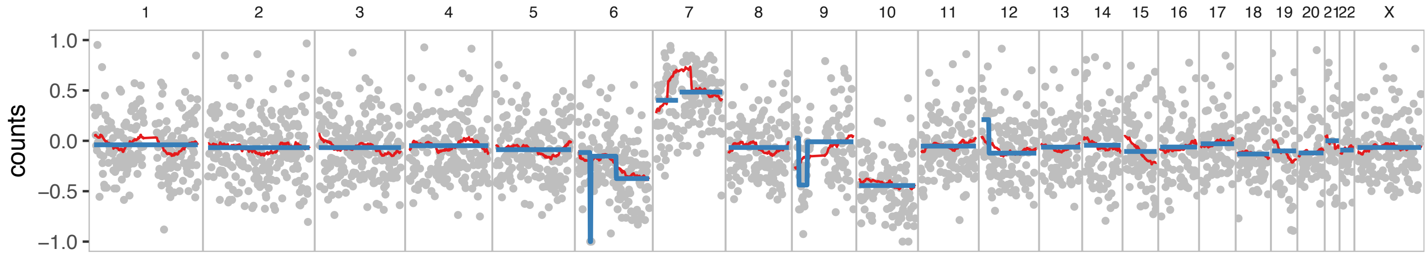

Methylation based classification

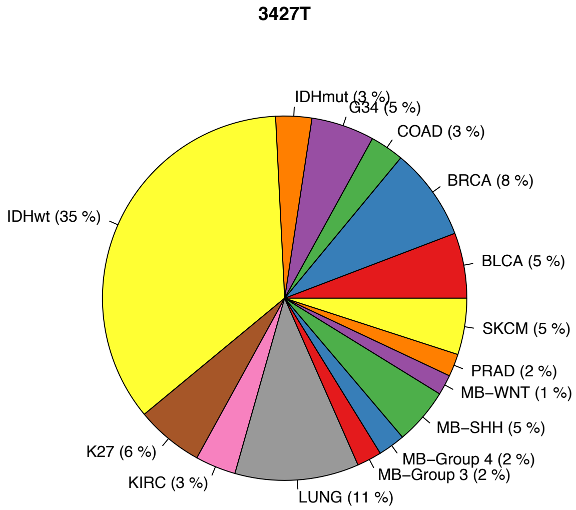

3523T

Quality control metrics

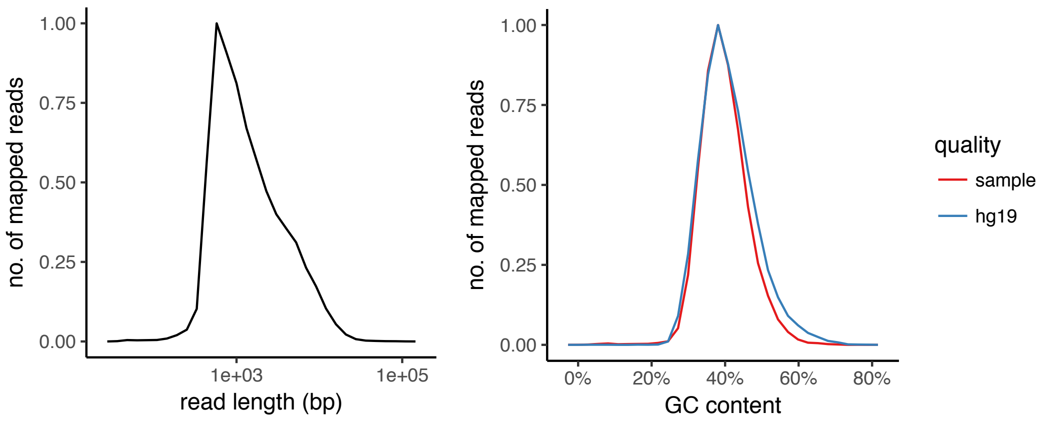

Copy number profile

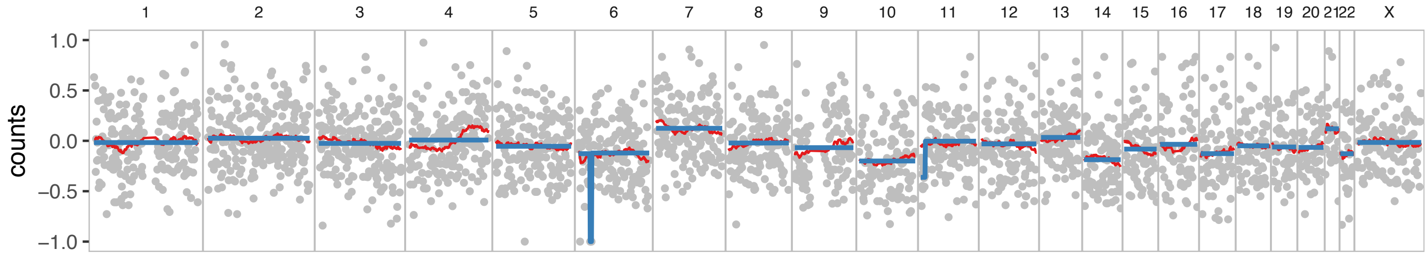

Methylation based classification

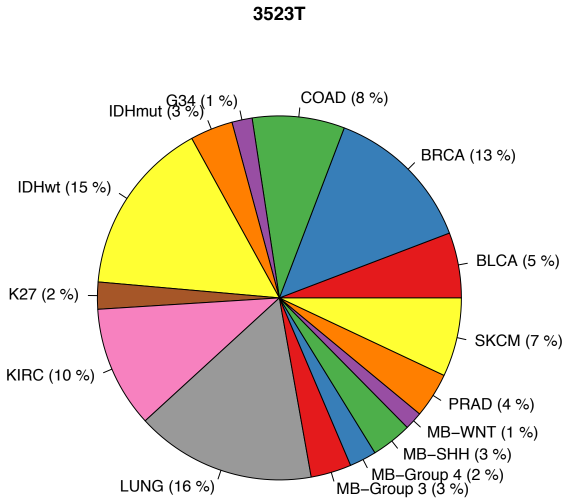

4596TP

Quality control metrics

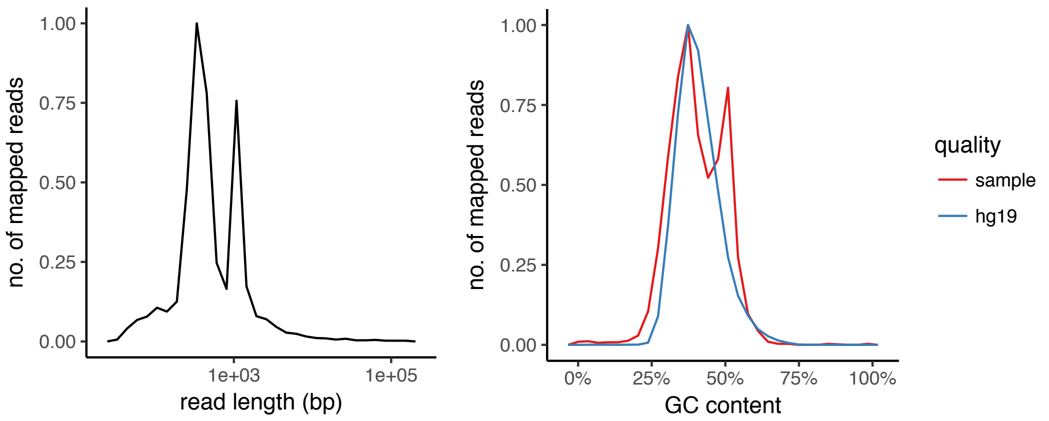

Copy number profile

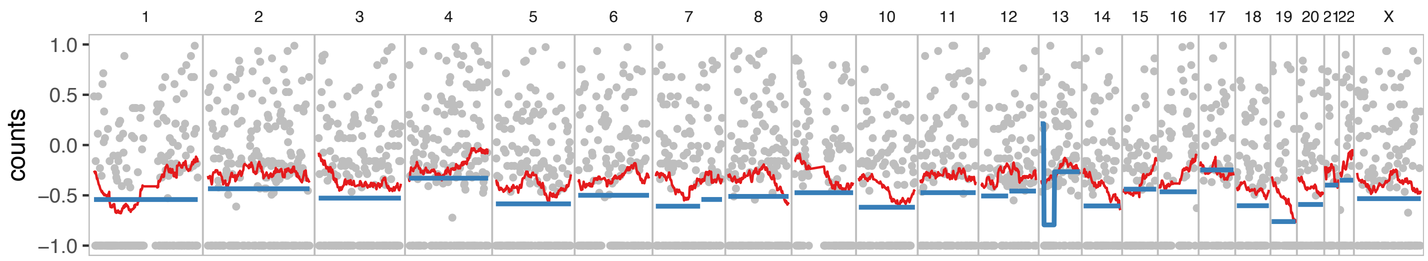

Methylation based classification

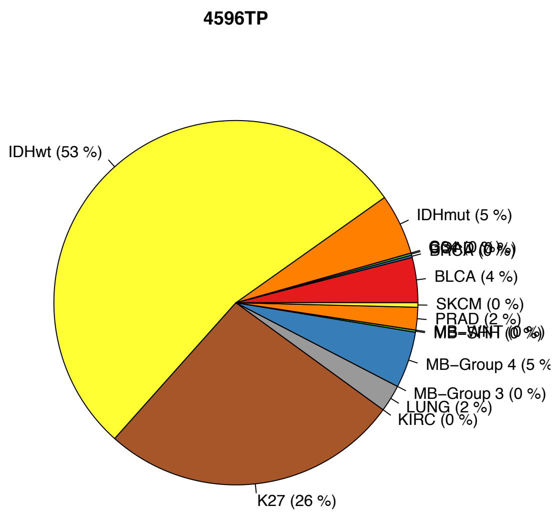

5337T

Quality control metrics

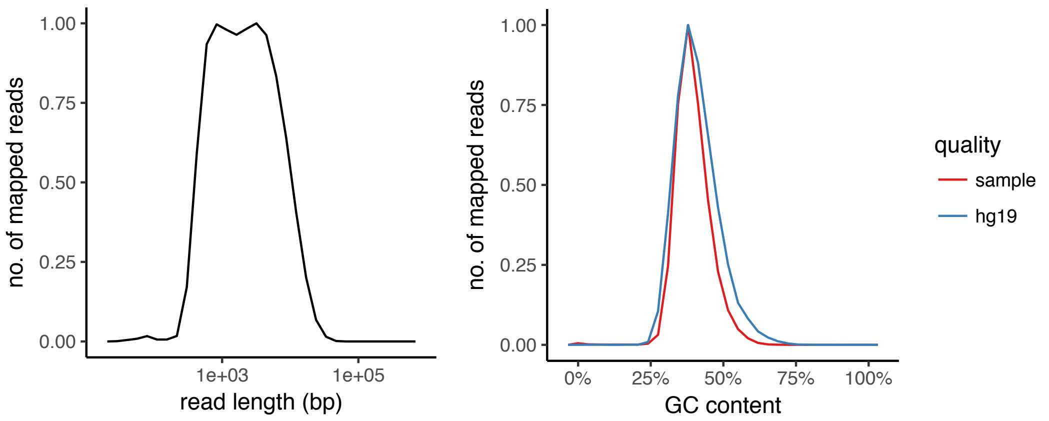

Copy number profile

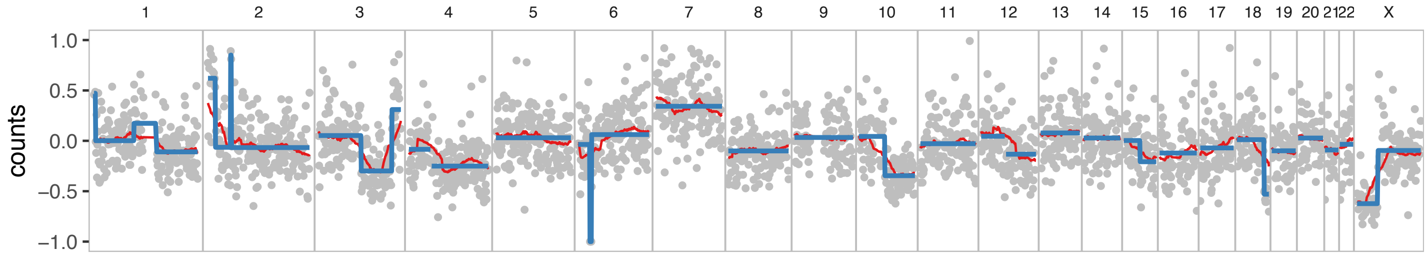

Methylation based classification

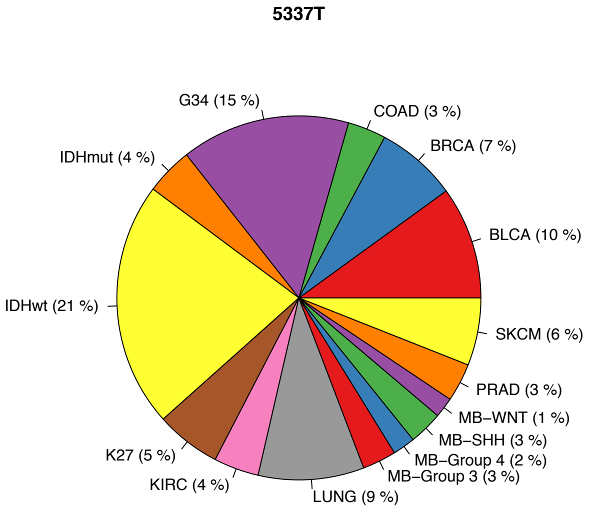

6228T

Quality control metrics

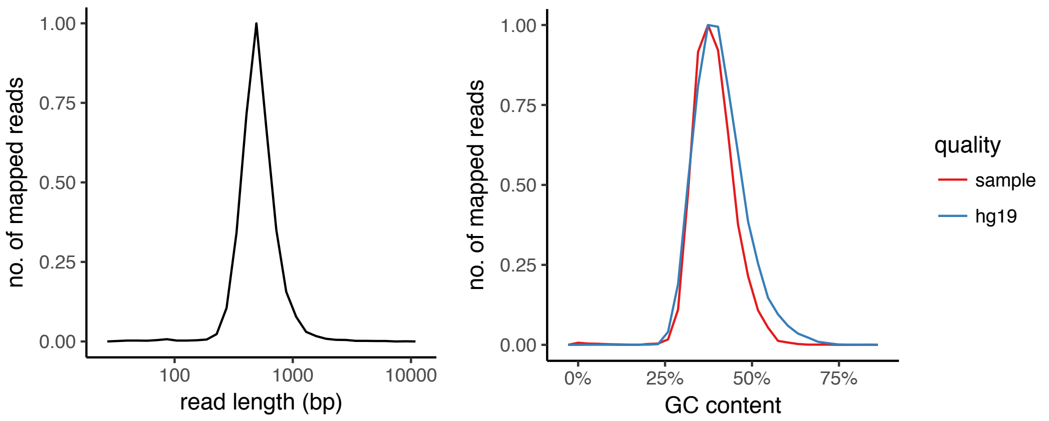

Copy number profile

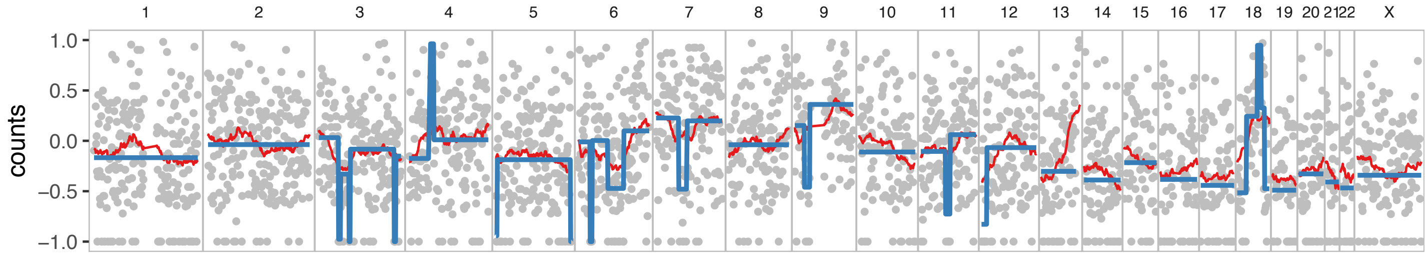

Methylation based classification

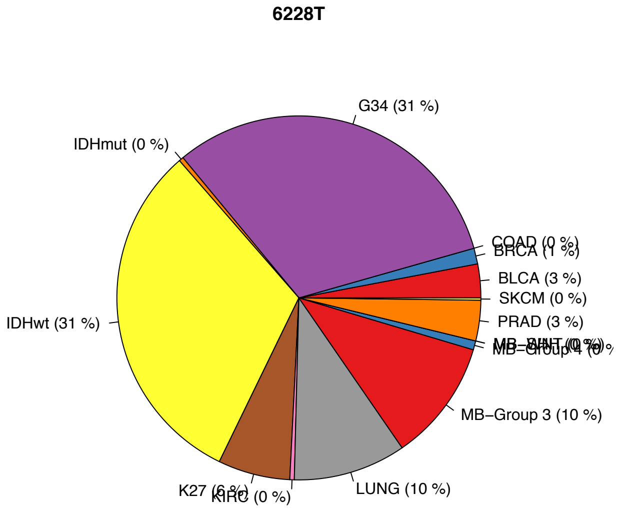

7382T

Quality control metrics

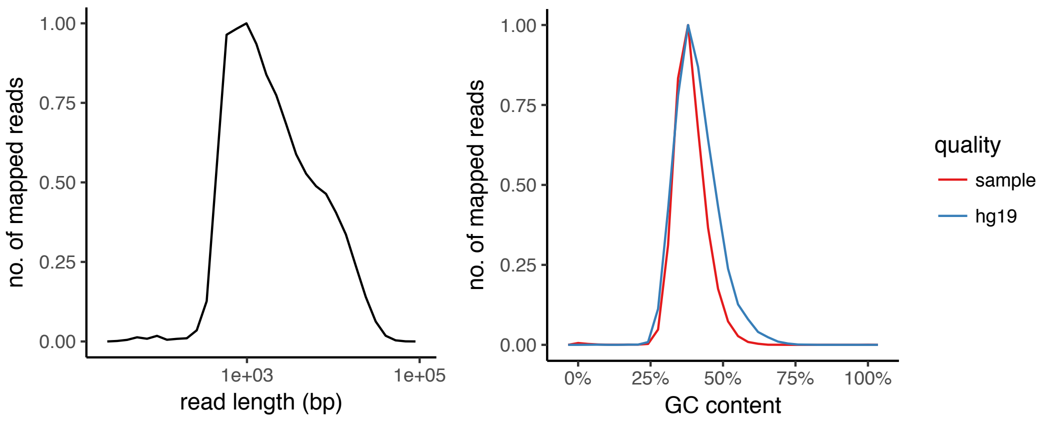

Copy number profile

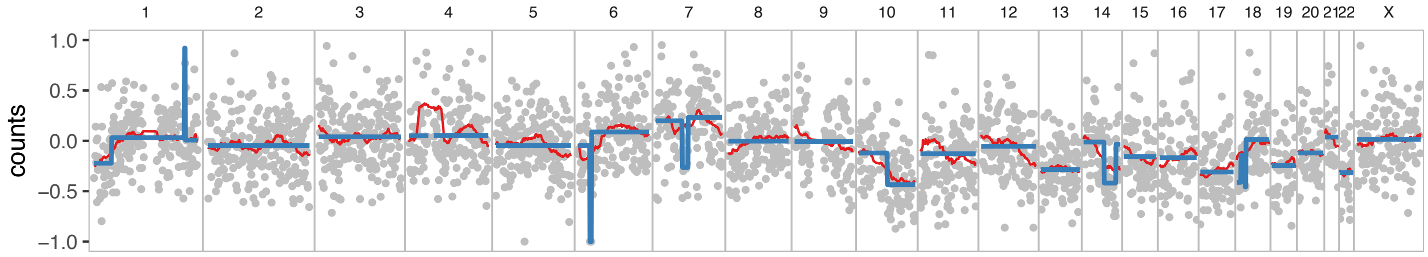

Methylation based classification

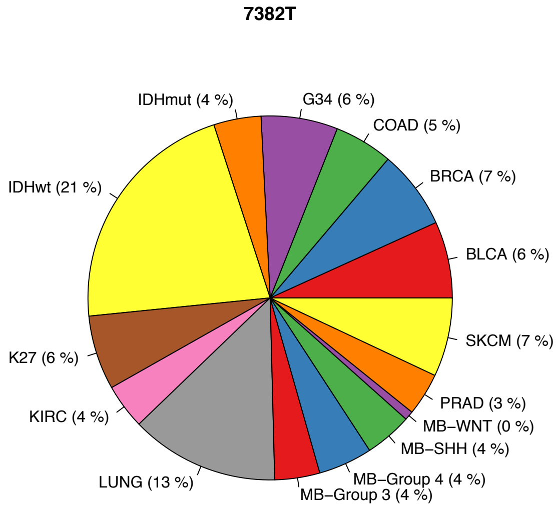

7455T

Quality control metrics

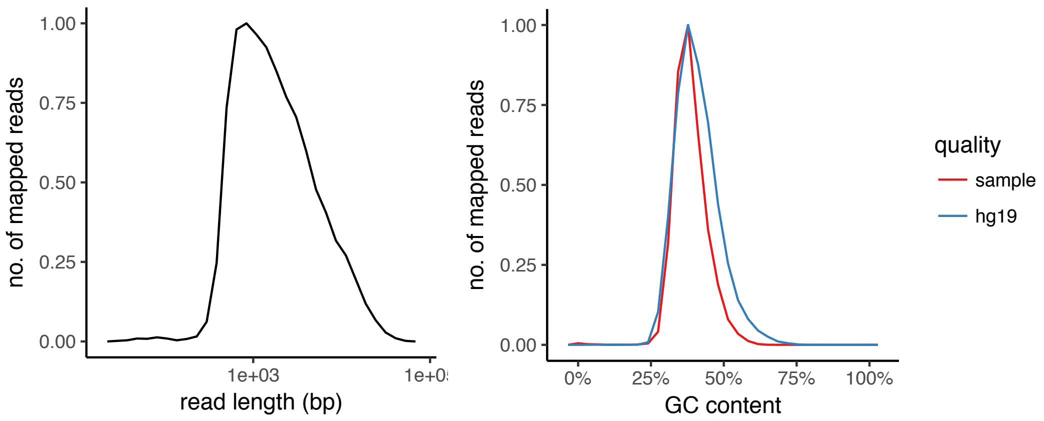

Copy number profile

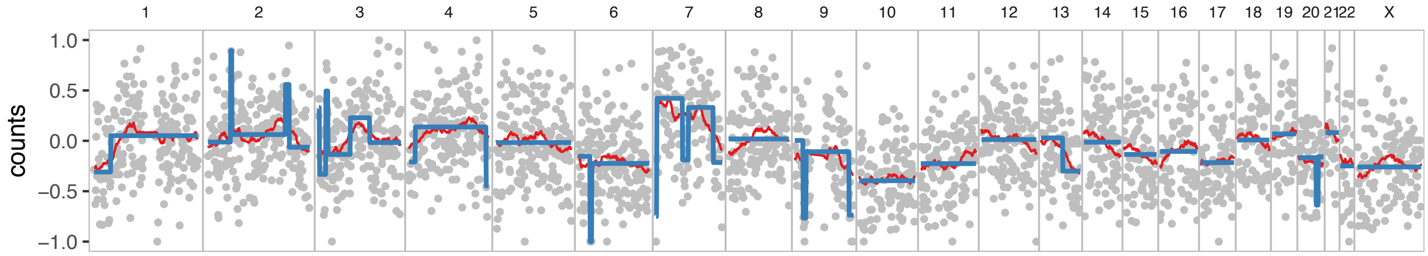

Methylation based classification

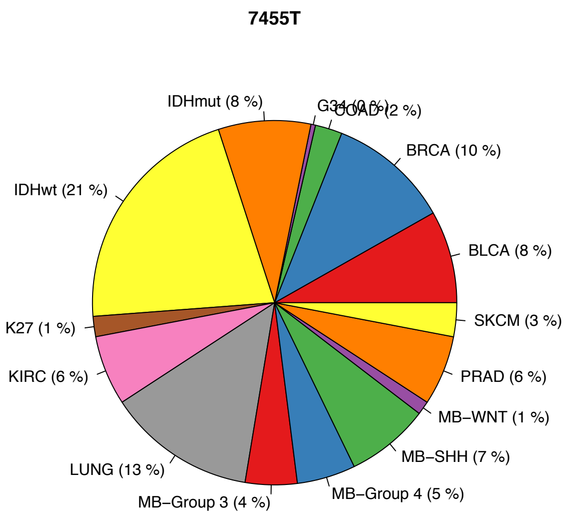

8137T

Quality control metrics

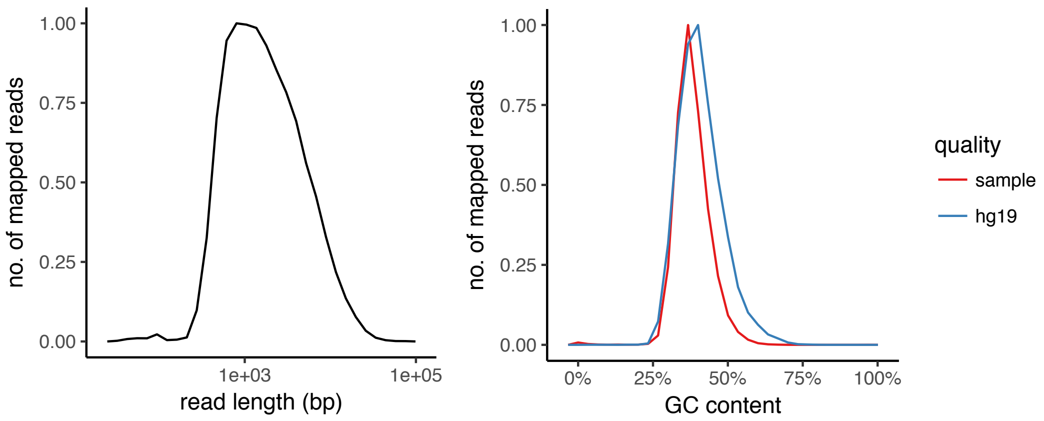

Copy number profile

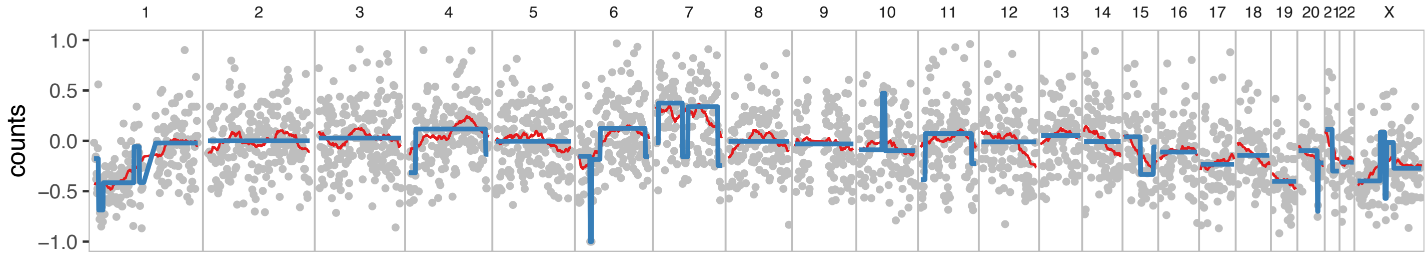

Methylation based classification

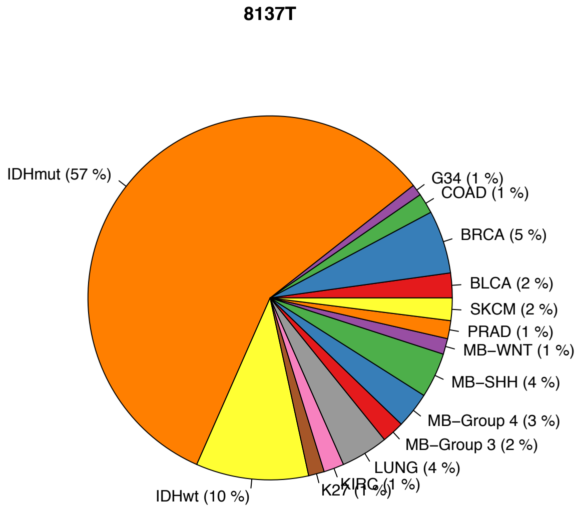

8146T

Quality control metrics

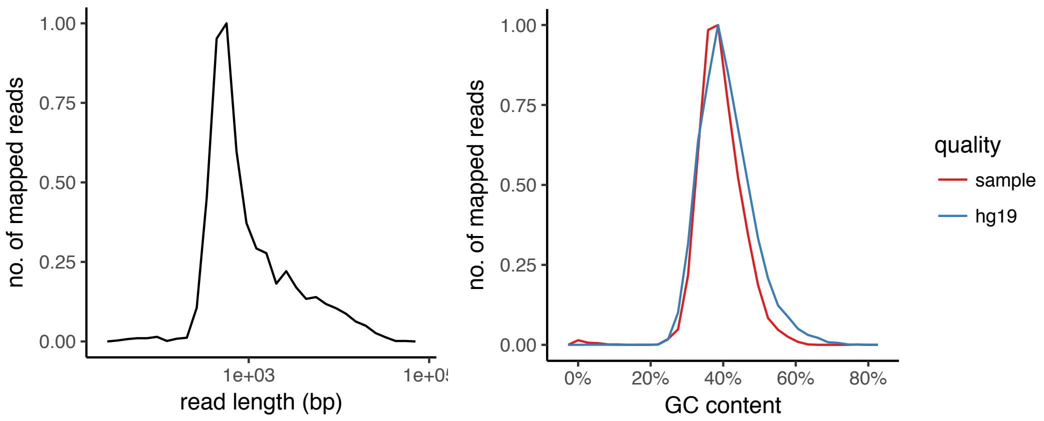

Copy number profile

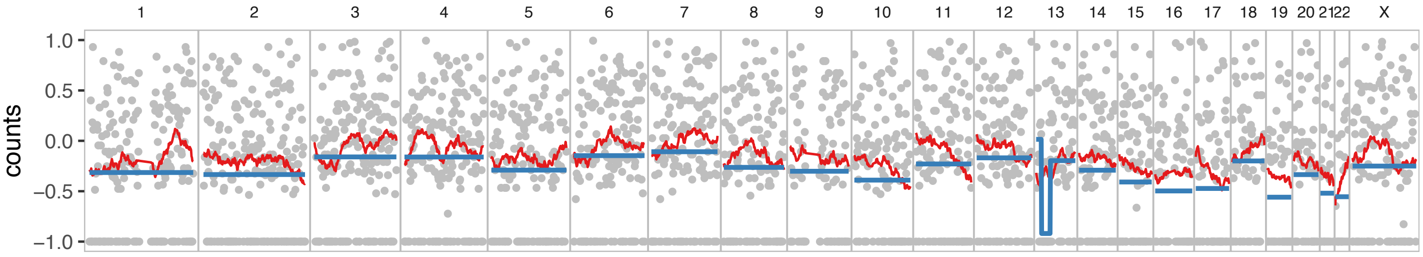

Methylation based classification

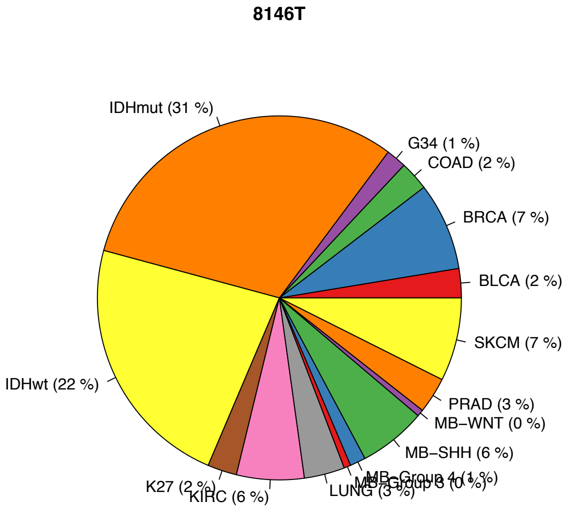

8355T

Quality control metrics

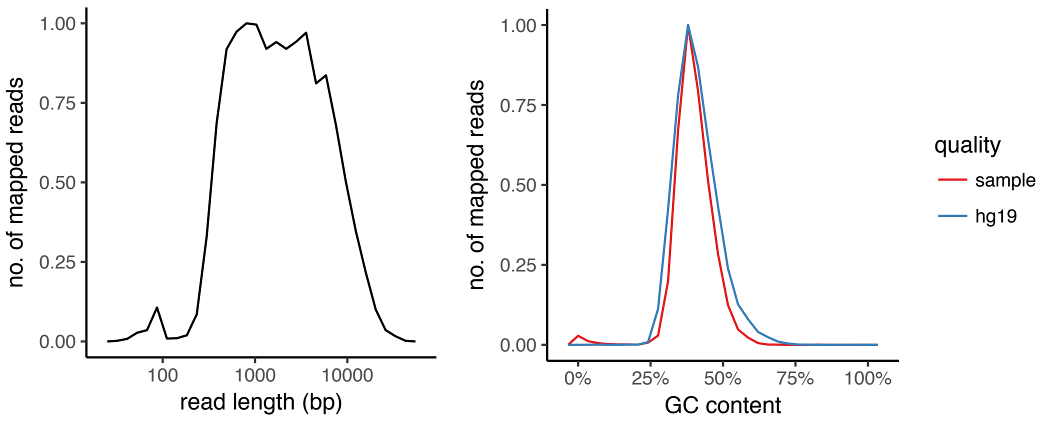

Copy number profile

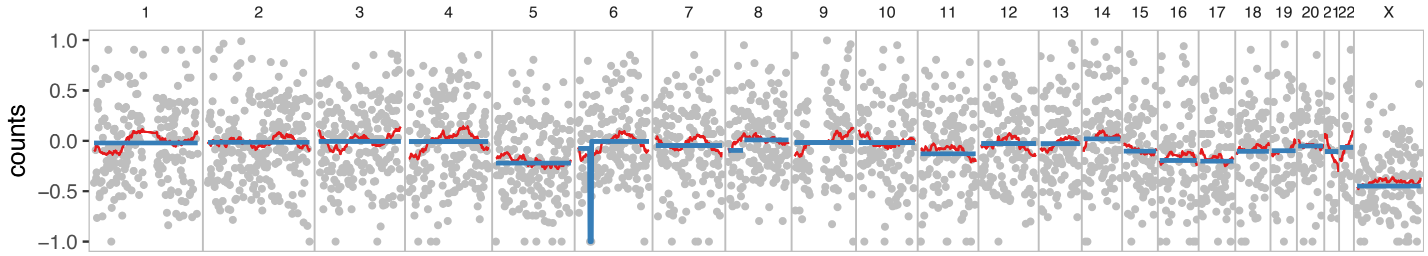

Methylation based classification

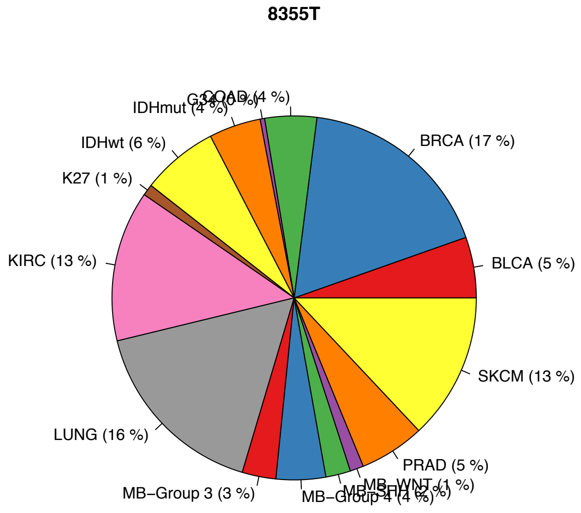

8356T

Quality control metrics

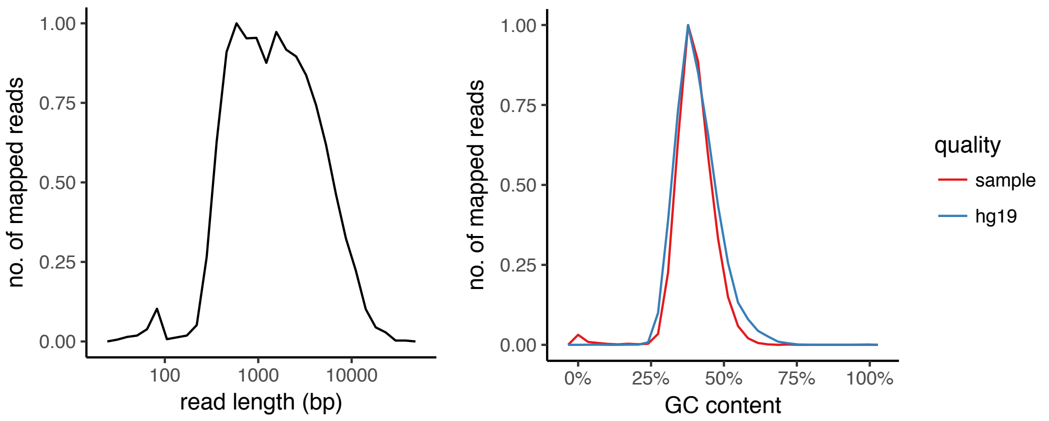

Copy number profile

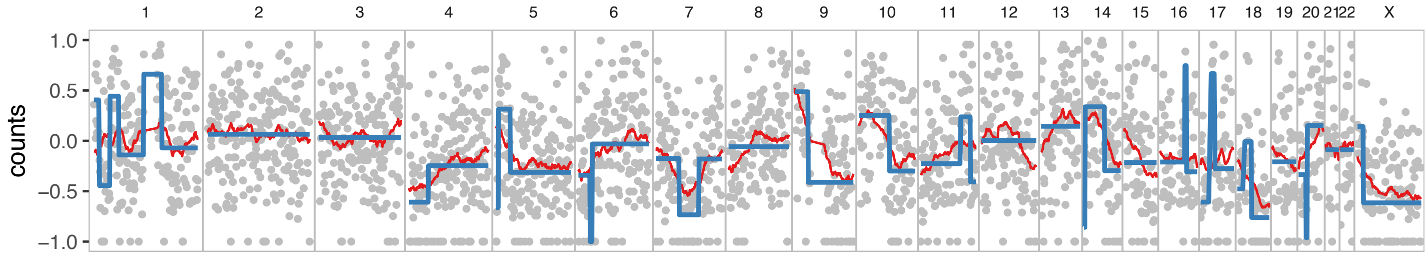

Methylation based classification

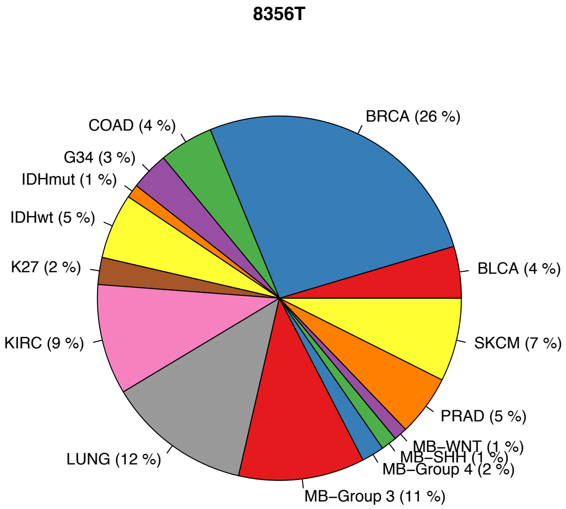

8357T

Quality control metrics

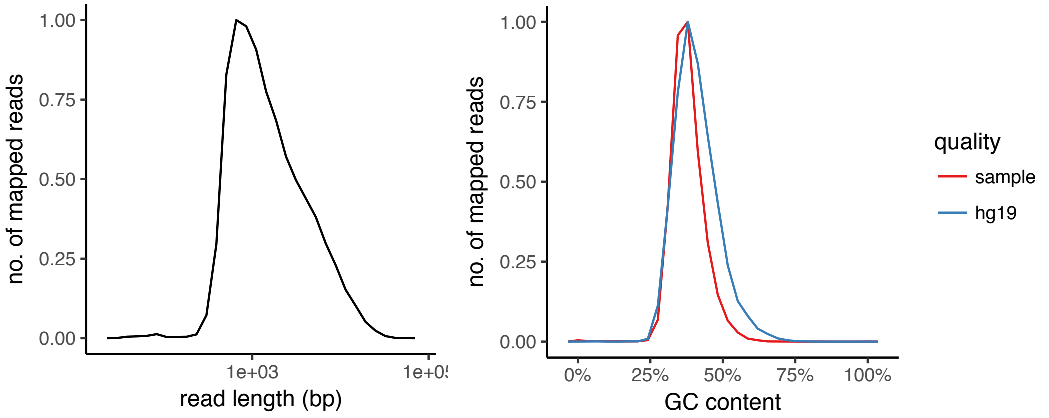

Copy number profile

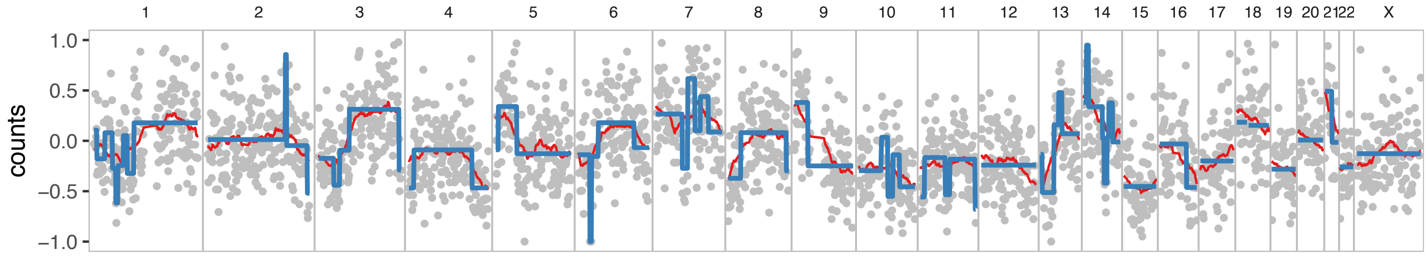

Methylation based classification

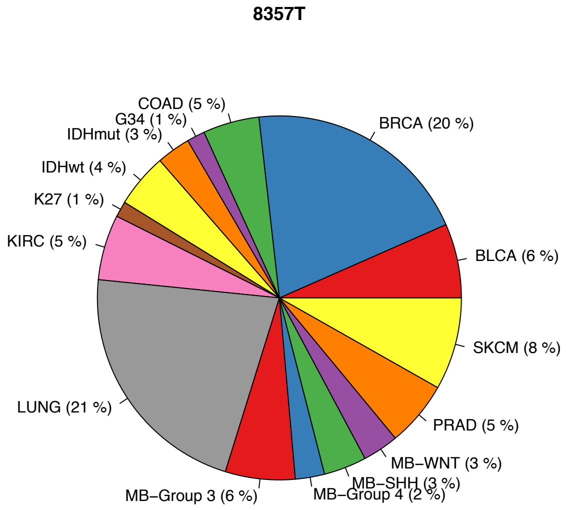

8358T

Quality control metrics

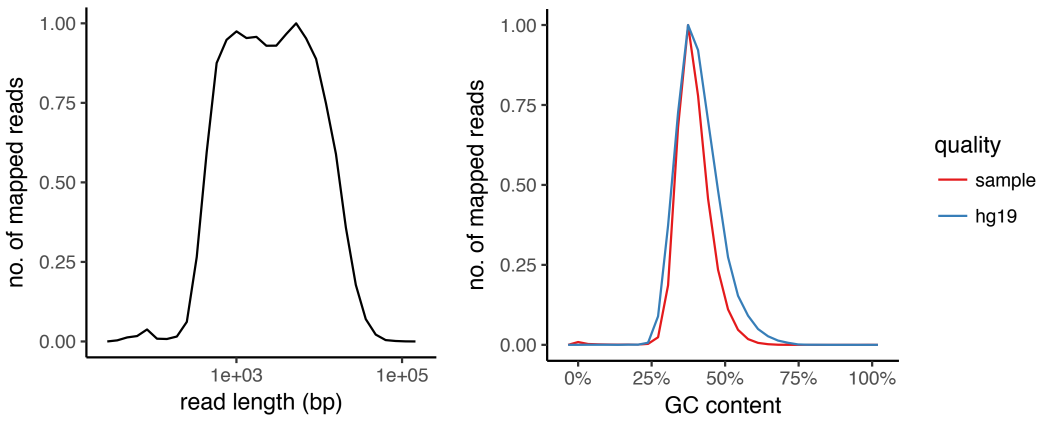

Copy number profile

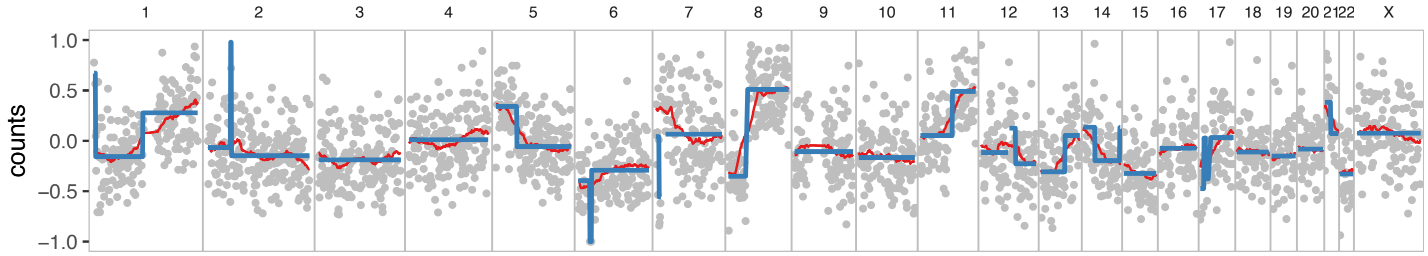

Methylation based classification

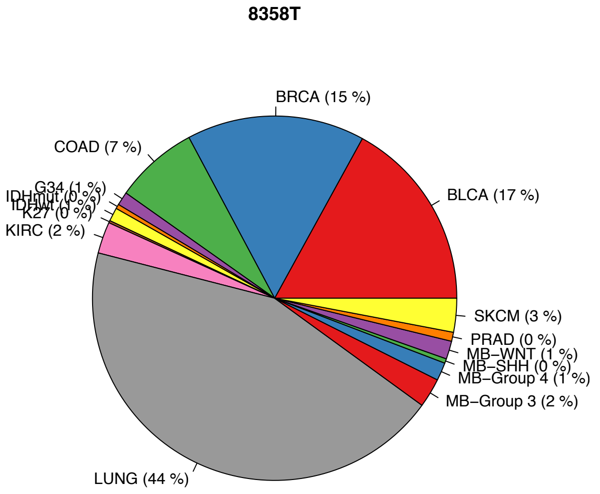

8359T

Quality control metrics

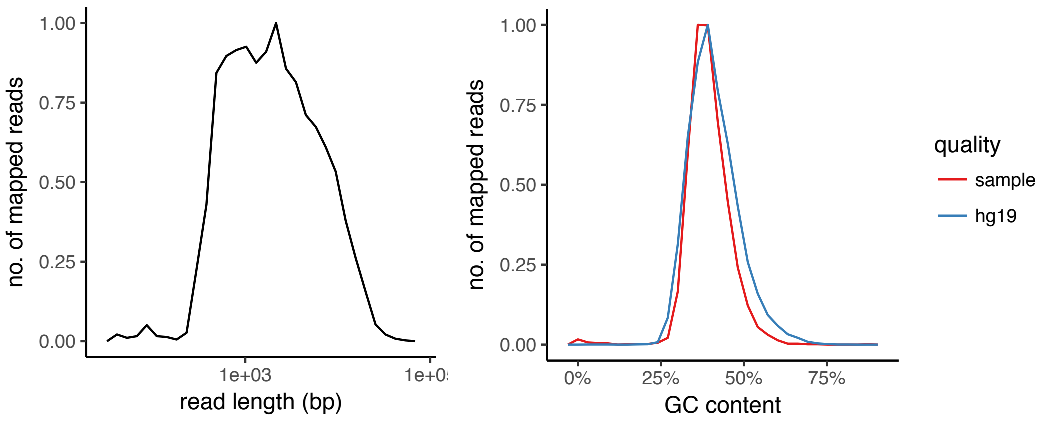

Copy number profile

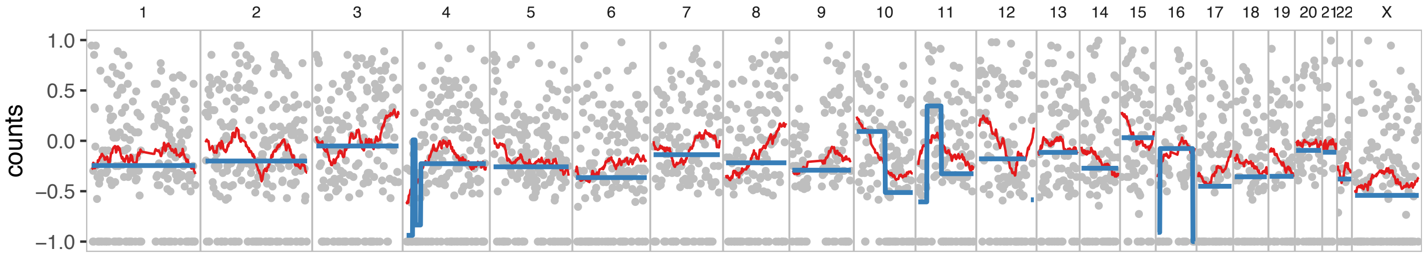

Methylation based classification

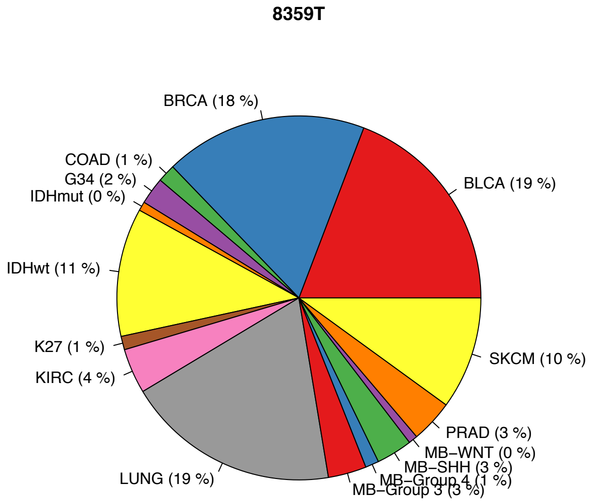

8372T

Quality control metrics

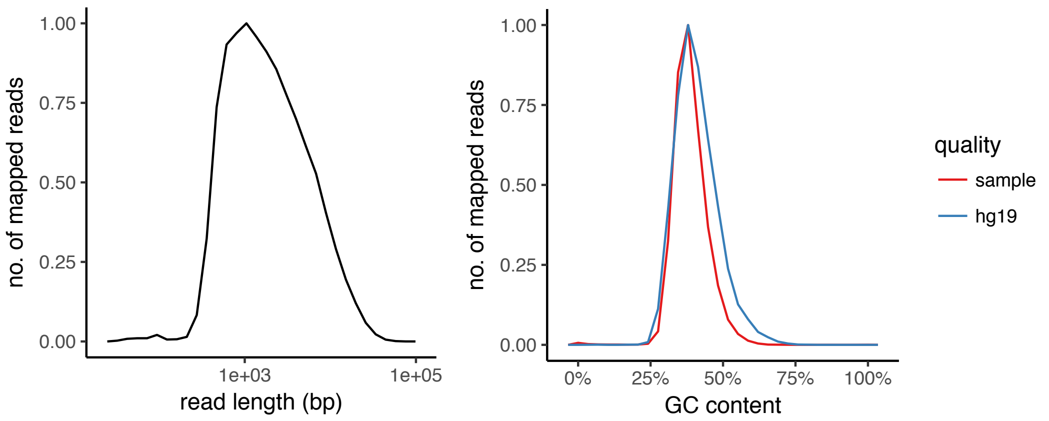

Copy number profile

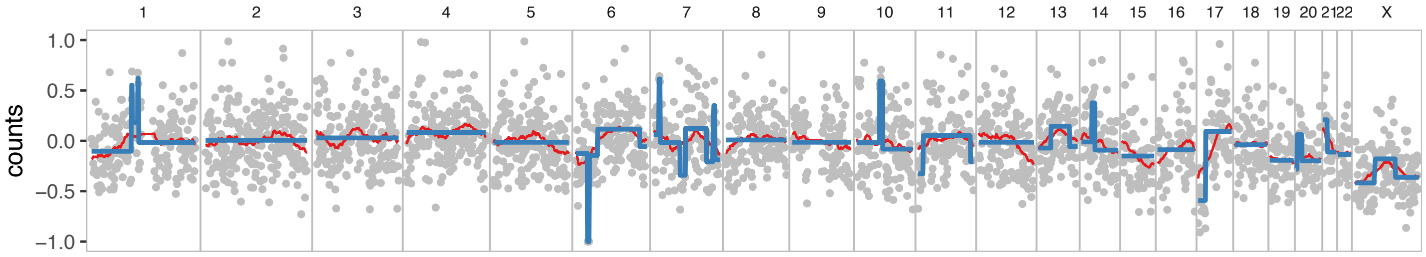

Methylation based classification

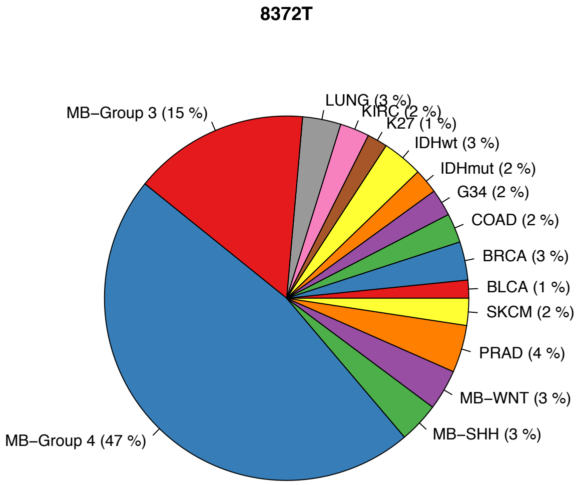

MB683

Quality control metrics

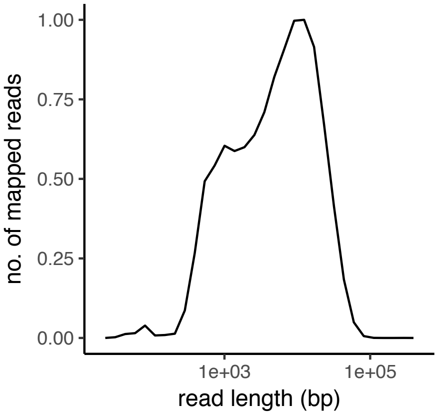

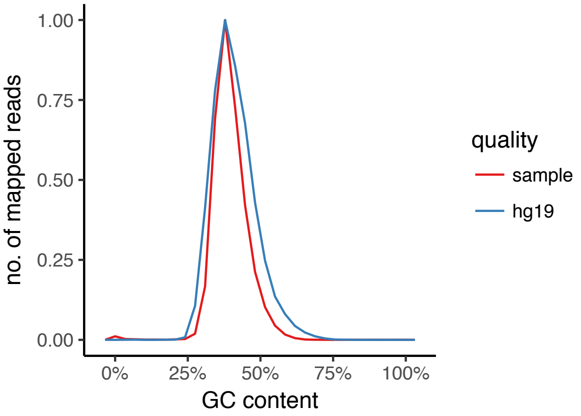

Copy number profile

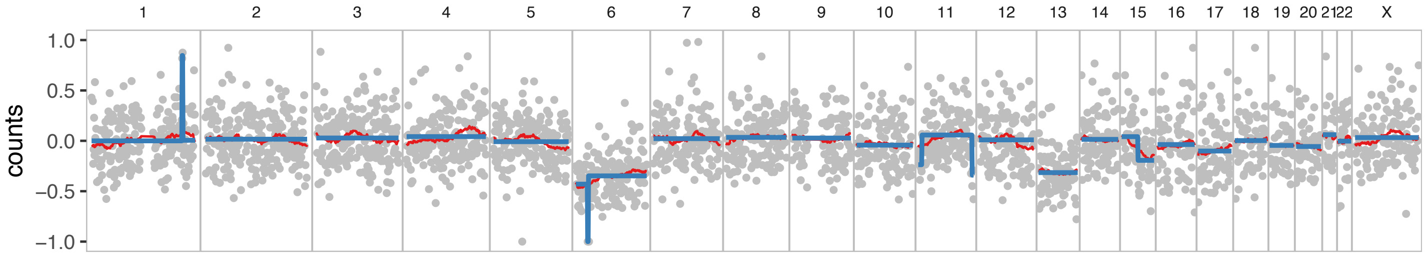

Methylation based classification

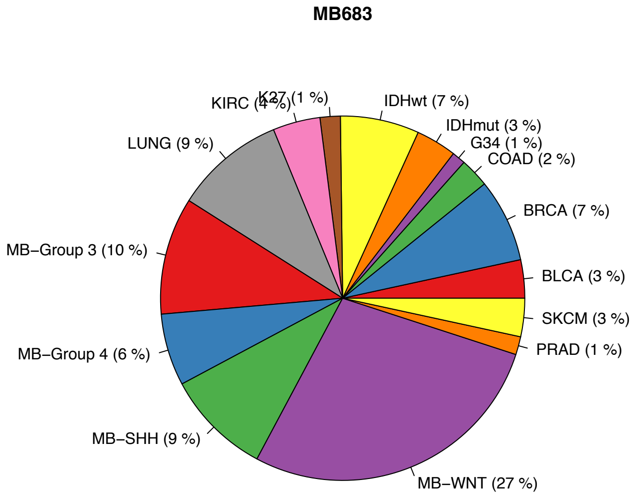

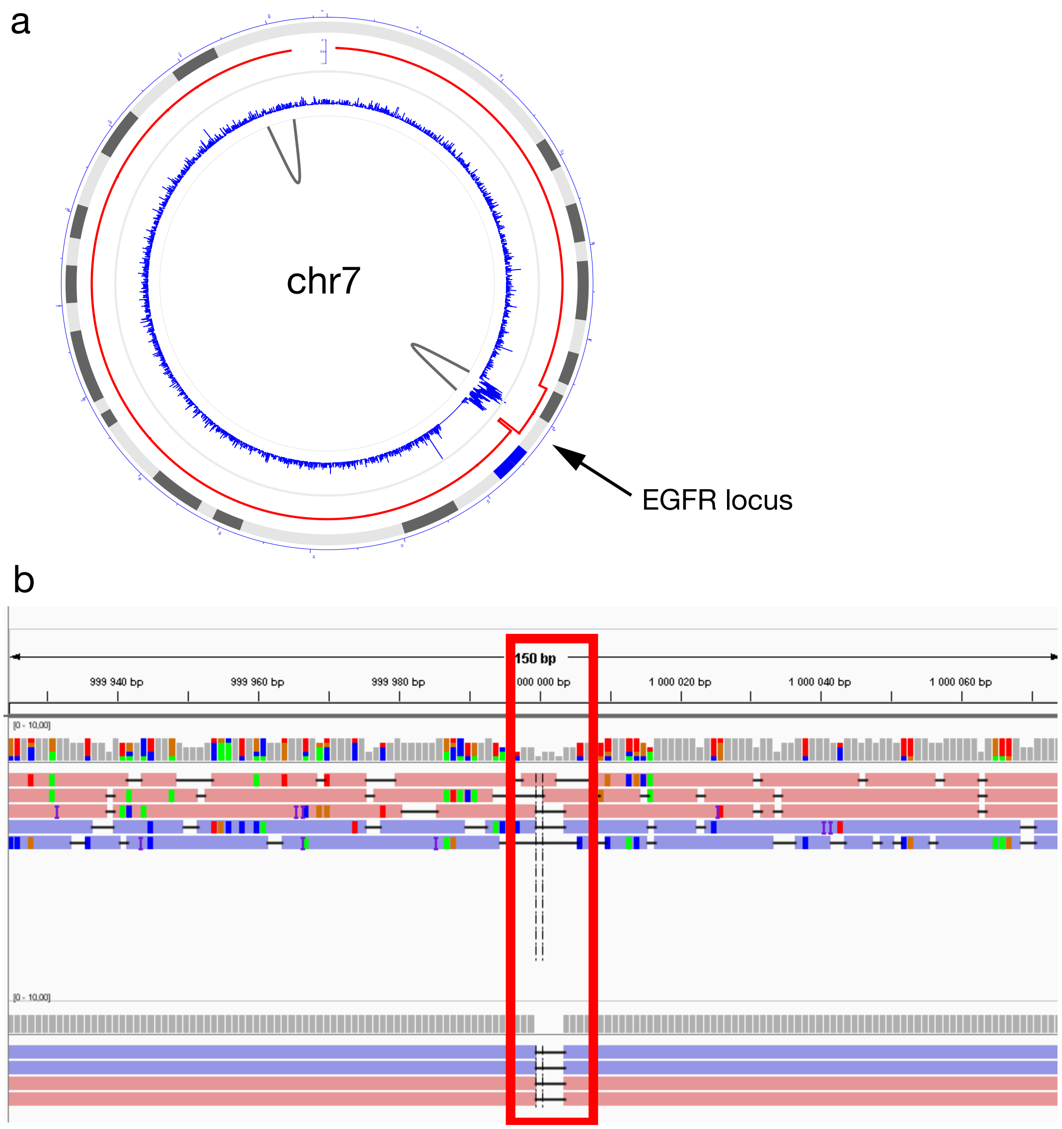

Fig. S2: Breakpoint identification for an EGFR double minute. (**a**) Circular plot of CNV and structural alteration on chromosome 7 in a glioblastoma (3427T) sample identified by NanoSV. Only CNVs larger than 1000bp are shown. Circular tracks from outside to inside: chromosome 7 cytobands (*black, grey*) with centromeric region (*blue*), CNVs log ratio values (red line), depth of coverage (*blue*), intrachromosomal structural alterations (*grey*). (**b**) Sanger sequencing of a PCR amplicon designed to circularization site confirms the predicted breakpoint (highlighted in *red*).

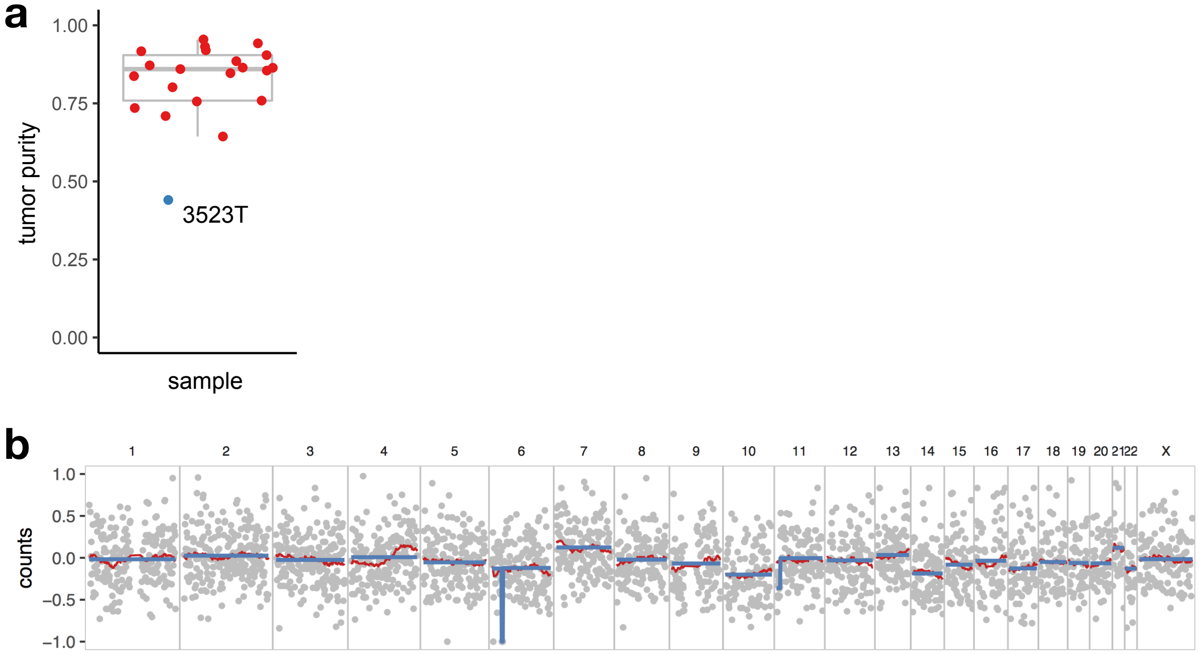

Fig. S3: Tumor purity estimation and influence on copy number profiling. (a) Tumor purity estimates from matched RNA-seq gene expression data using ESTIMATE *(12)* identifies low purity for sample 3523T in comparison to a series of GBM from the same institution *(7)*. **(b)** CN profile for sample 3523T shows correct segmentation (blue) but low amplitude of log ratios. Automatic CN calling using fixed thresholds thus missed chromosome 7 gain and chromosome 10 loss. Red lines indicate running mean of log ratios.

Fig. S4: Sequencing reports for nanopore amplicon sequencing. For each sample, we report read depth (no. of reads) per targeted genomic region (*colored*) over the time frame of the entire sequencing run. When minimum coverage of 1000X in all amplicons could be achieved, the time to reach 1000X depth is indicated.

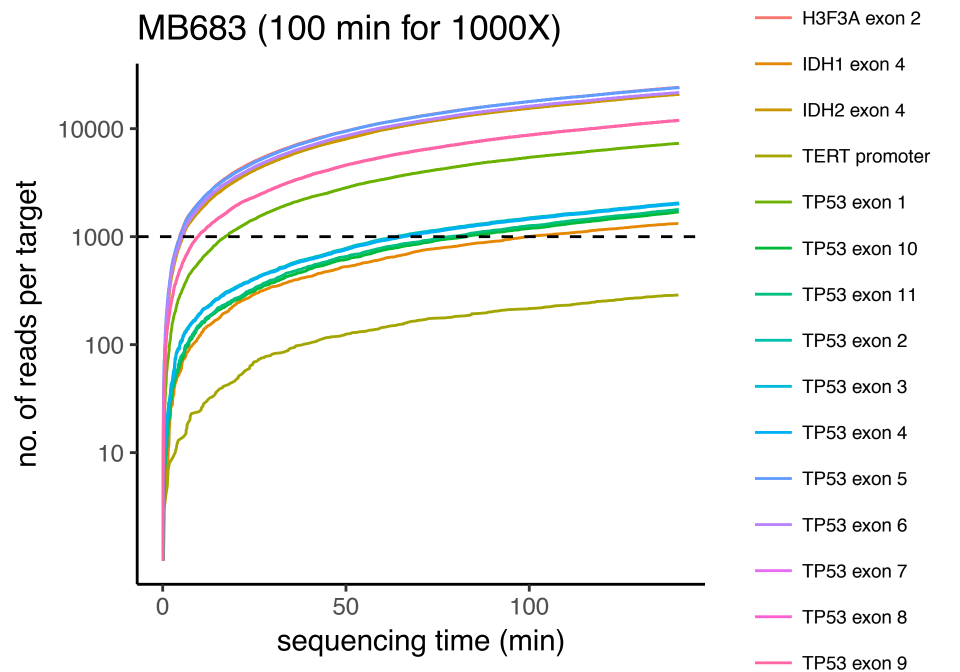
MB683 (100 min for 1000X)
no. of reads per target
10000
1000
100
10
0
50
100
sequencing time (min)
H3F3A exon 2
IDH1 exon 4
IDH2 exon 4
TERT promoter
TP53 exon 1
TP53 exon 10
TP53 exon 11
TP53 exon 2
TP53 exon 3
TP53 exon 4
TP53 exon 5
TP53 exon 6
TP53 exon 7
TP53 exon 8
TP53 exon 9

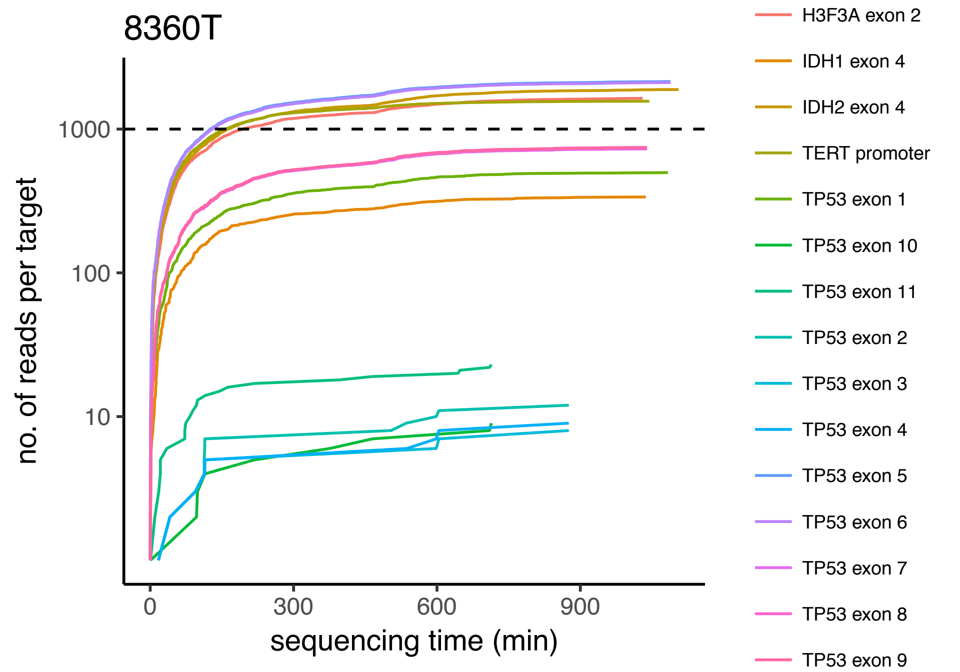
8360T
1000
100
10
no. of reads per target
0
300
600
900
sequencing time (min)
H3F3A exon 2
IDH1 exon 4
IDH2 exon 4
TERT promoter
TP53 exon 1
TP53 exon 10
TP53 exon 11
TP53 exon 2
TP53 exon 3
TP53 exon 4
TP53 exon 5
TP53 exon 6
TP53 exon 7
TP53 exon 8
TP53 exon 9

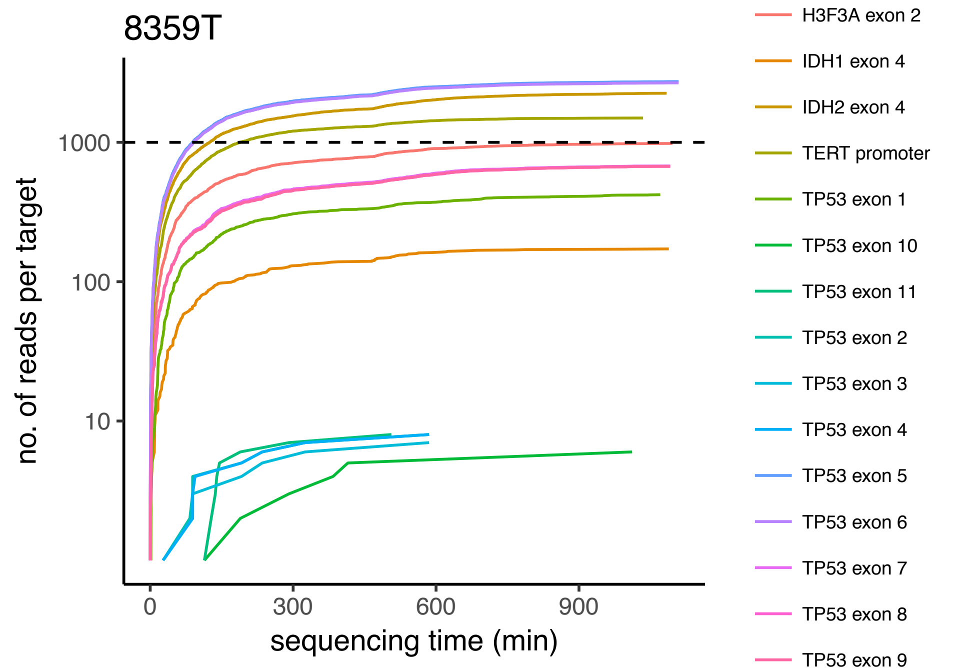

8359T
no. of reads per target
1000
100
10
0
300
600
900
sequencing time (min)
H3F3A exon 2
IDH1 exon 4
IDH2 exon 4
TERT promoter
TP53 exon 1
TP53 exon 10
TP53 exon 11
TP53 exon 2
TP53 exon 3
TP53 exon 4
TP53 exon 5
TP53 exon 6
TP53 exon 7
TP53 exon 8
TP53 exon 9

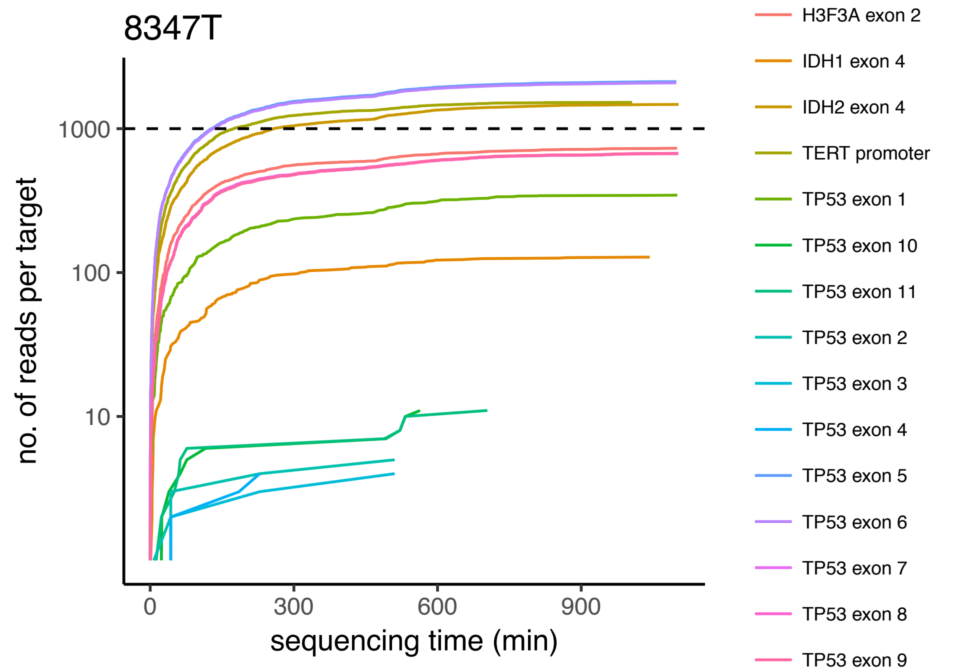

8347T
no. of reads per target
1000
100
10
0
300
600
900
sequencing time (min)
H3F3A exon 2
IDH1 exon 4
IDH2 exon 4
TERT promoter
TP53 exon 1
TP53 exon 10
TP53 exon 11
TP53 exon 2
TP53 exon 3
TP53 exon 4
TP53 exon 5
TP53 exon 6
TP53 exon 7
TP53 exon 8
TP53 exon 9

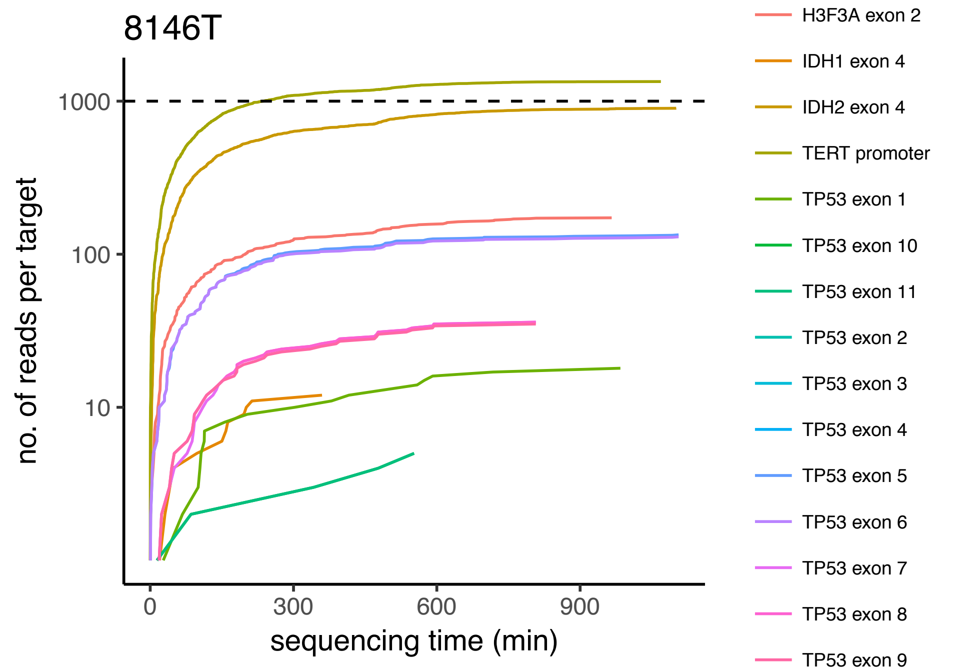
8146T
no. of reads per target
1000
100
10
0
300
600
900
sequencing time (min)
H3F3A exon 2
IDH1 exon 4
IDH2 exon 4
TERT promoter
TP53 exon 1
TP53 exon 10
TP53 exon 11
TP53 exon 2
TP53 exon 3
TP53 exon 4
TP53 exon 5
TP53 exon 6
TP53 exon 7
TP53 exon 8
TP53 exon 9

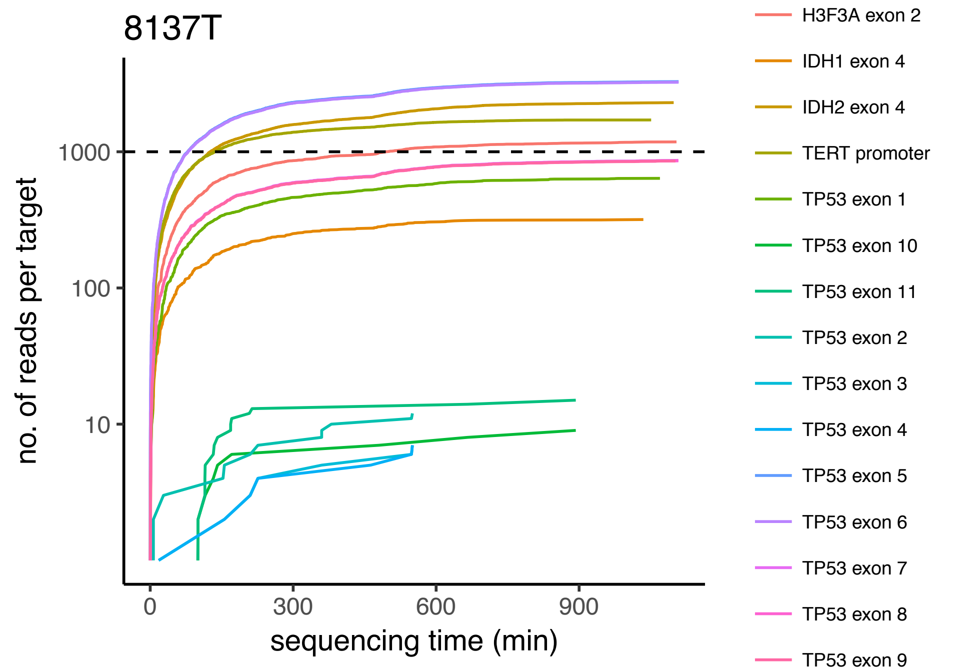
8137T
no. of reads per target
1000
100
10
0
300
600
900
sequencing time (min)
H3F3A exon 2
IDH1 exon 4
IDH2 exon 4
TERT promoter
TP53 exon 1
TP53 exon 10
TP53 exon 11
TP53 exon 2
TP53 exon 3
TP53 exon 4
TP53 exon 5
TP53 exon 6
TP53 exon 7
TP53 exon 8
TP53 exon 9

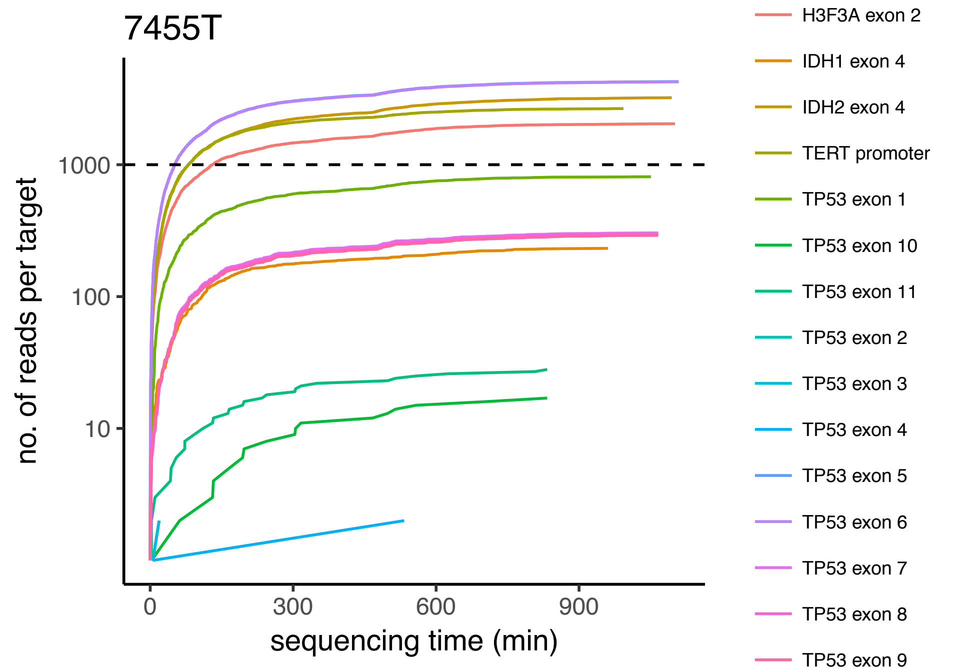
7455T
no. of reads per target
1000
100
10
0
300
600
900
sequencing time (min)
H3F3A exon 2
IDH1 exon 4
IDH2 exon 4
TERT promoter
TP53 exon 1
TP53 exon 10
TP53 exon 11
TP53 exon 2
TP53 exon 3
TP53 exon 4
TP53 exon 5
TP53 exon 6
TP53 exon 7
TP53 exon 8
TP53 exon 9

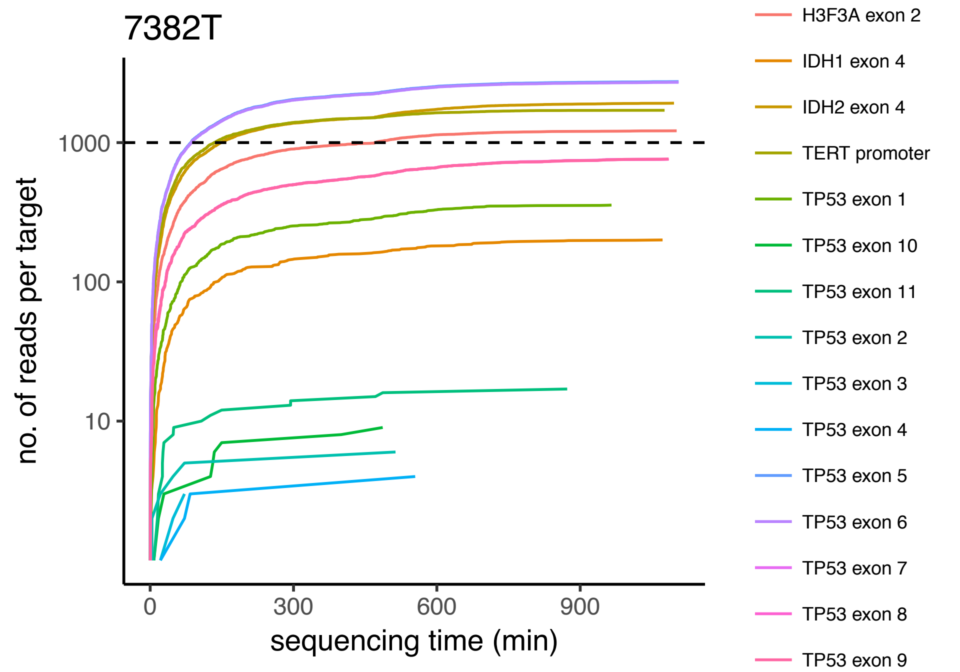
7382T
no. of reads per target
1000
100
10
0
300
600
900
sequencing time (min)
H3F3A exon 2
IDH1 exon 4
IDH2 exon 4
TERT promoter
TP53 exon 1
TP53 exon 10
TP53 exon 11
TP53 exon 2
TP53 exon 3
TP53 exon 4
TP53 exon 5
TP53 exon 6
TP53 exon 7
TP53 exon 8
TP53 exon 9

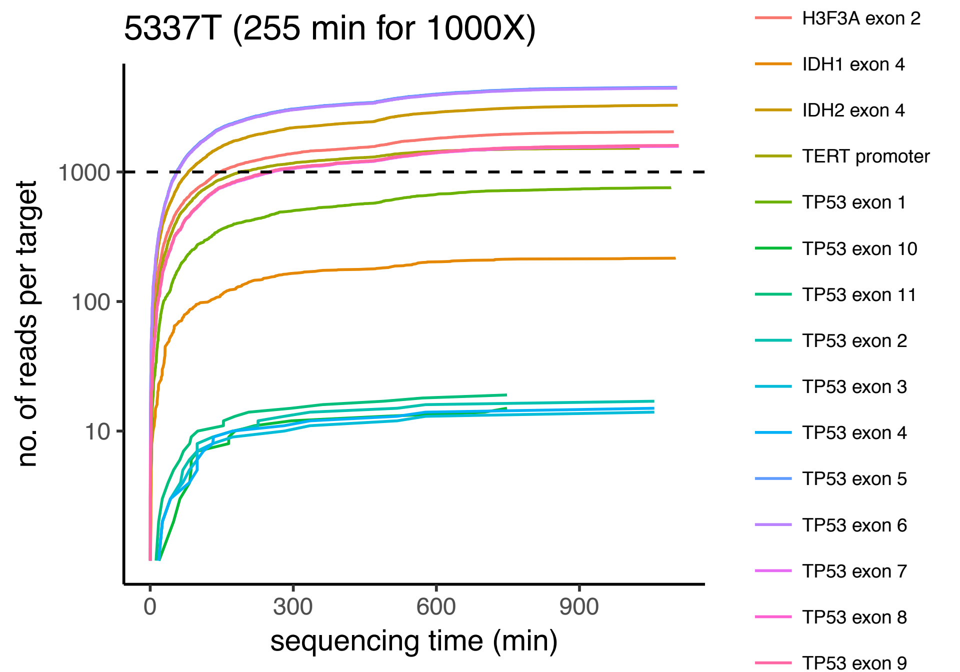

5337T (255 min for 1000X)
no. of reads per target
1000
100
10
0
300
600
900
sequencing time (min)
H3F3A exon 2
IDH1 exon 4
IDH2 exon 4
TERT promoter
TP53 exon 1
TP53 exon 10
TP53 exon 11
TP53 exon 2
TP53 exon 3
TP53 exon 4
TP53 exon 5
TP53 exon 6
TP53 exon 7
TP53 exon 8
TP53 exon 9

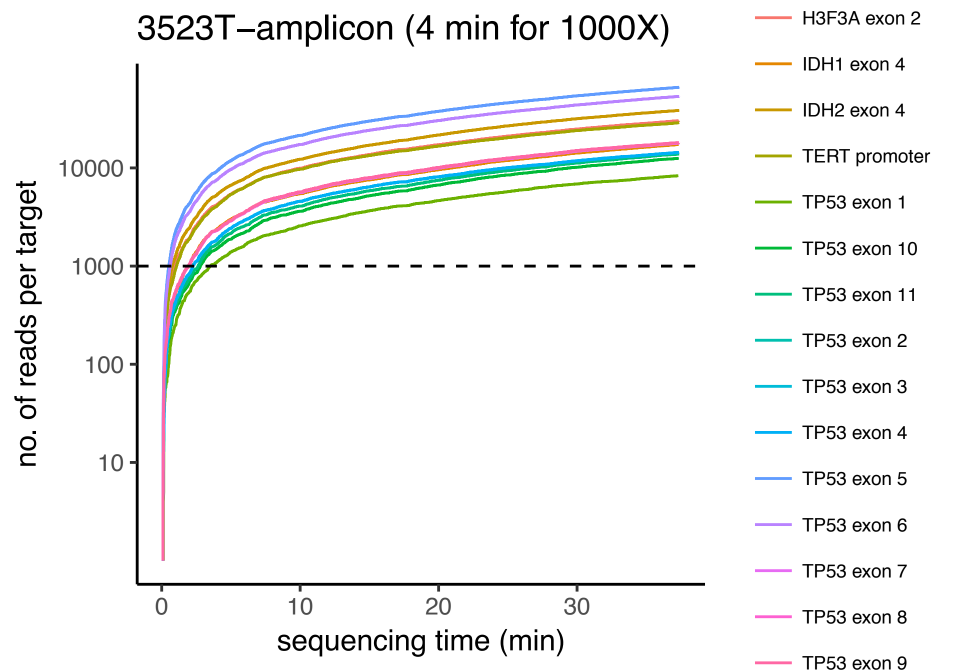
3523T–amplicon (4 min for 1000X)
no. of reads per target
10000
1000
100
10
0
10
20
30
sequencing time (min)
H3F3A exon 2
IDH1 exon 4
IDH2 exon 4
TERT promoter
TP53 exon 1
TP53 exon 10
TP53 exon 11
TP53 exon 2
TP53 exon 3
TP53 exon 4
TP53 exon 5
TP53 exon 6
TP53 exon 7
TP53 exon 8
TP53 exon 9

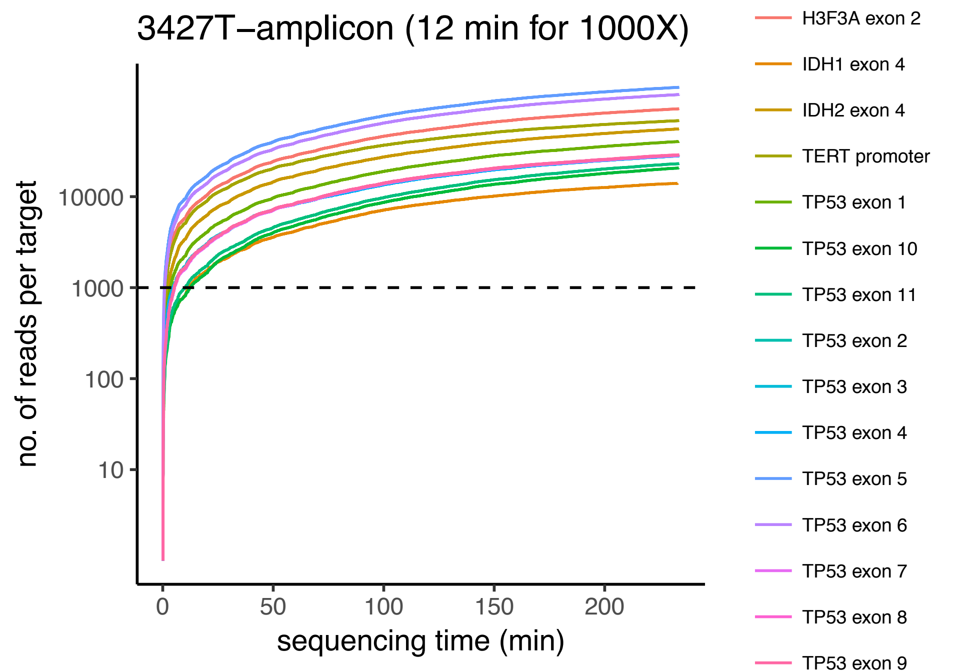
3427T–amplicon (12 min for 1000X)
no. of reads per target
10000
1000
100
10
0
50
100
150
200
sequencing time (min)
H3F3A exon 2
IDH1 exon 4
IDH2 exon 4
TERT promoter
TP53 exon 1
TP53 exon 10
TP53 exon 11
TP53 exon 2
TP53 exon 3
TP53 exon 4
TP53 exon 5
TP53 exon 6
TP53 exon 7
TP53 exon 8
TP53 exon 9

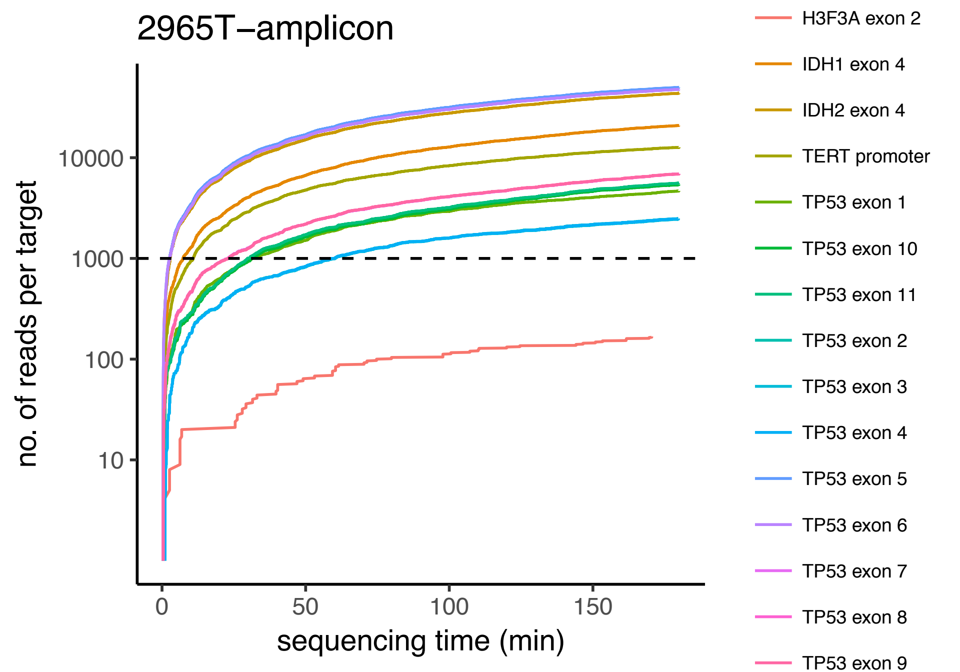

2965T−amplicon
no. of reads per target
10000
1000
100
10
0
50
100
150
sequencing time (min)
H3F3A exon 2
IDH1 exon 4
IDH2 exon 4
TERT promoter
TP53 exon 1
TP53 exon 10
TP53 exon 11
TP53 exon 2
TP53 exon 3
TP53 exon 4
TP53 exon 5
TP53 exon 6
TP53 exon 7
TP53 exon 8
TP53 exon 9

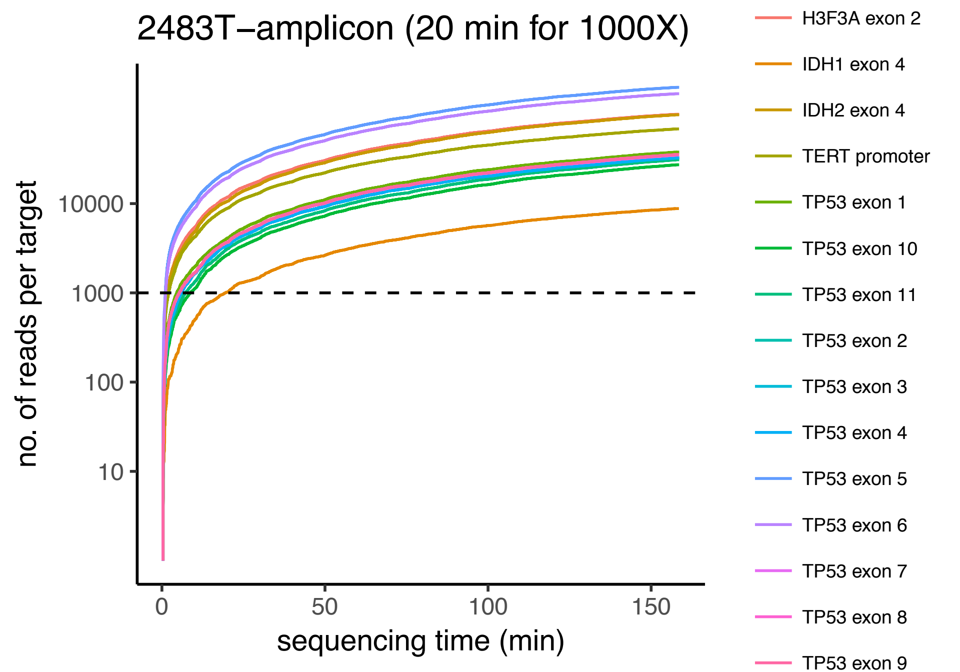
2483T–amplicon (20 min for 1000X)
no. of reads per target
10
100
1000
10000
0
50
100
150
sequencing time (min)
H3F3A exon 2
IDH1 exon 4
IDH2 exon 4
TERT promoter
TP53 exon 1
TP53 exon 10
TP53 exon 11
TP53 exon 2
TP53 exon 3
TP53 exon 4
TP53 exon 5
TP53 exon 6
TP53 exon 7
TP53 exon 8
TP53 exon 9

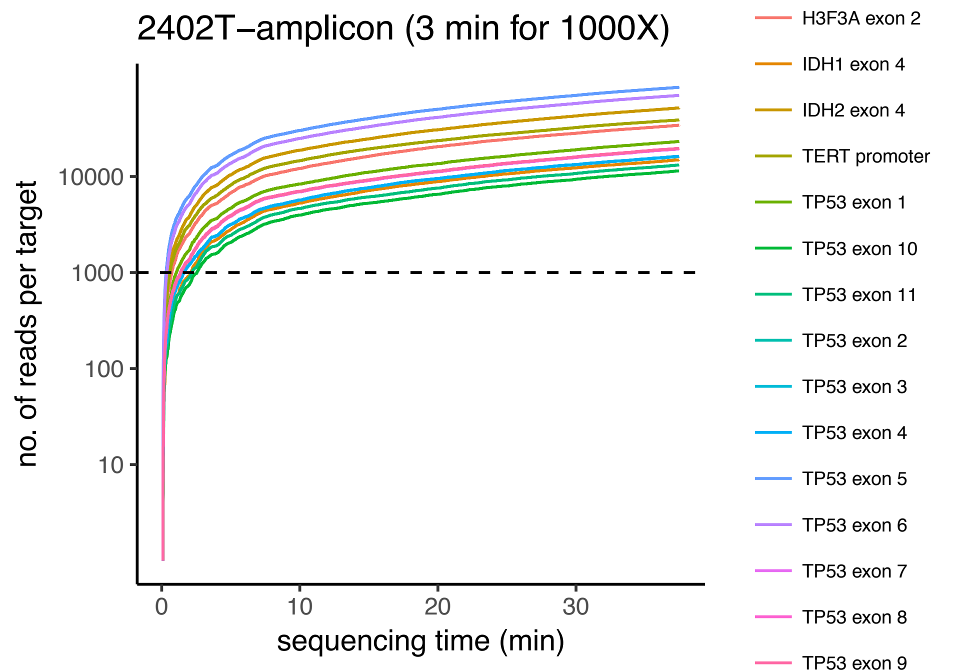
2402T−amplicon (3 min for 1000X)
no. of reads per target
10000
1000
100
10
0
10
20
30
sequencing time (min)
H3F3A exon 2
IDH1 exon 4
IDH2 exon 4
TERT promoter
TP53 exon 1
TP53 exon 10
TP53 exon 11
TP53 exon 2
TP53 exon 3
TP53 exon 4
TP53 exon 5
TP53 exon 6
TP53 exon 7
TP53 exon 8
TP53 exon 9

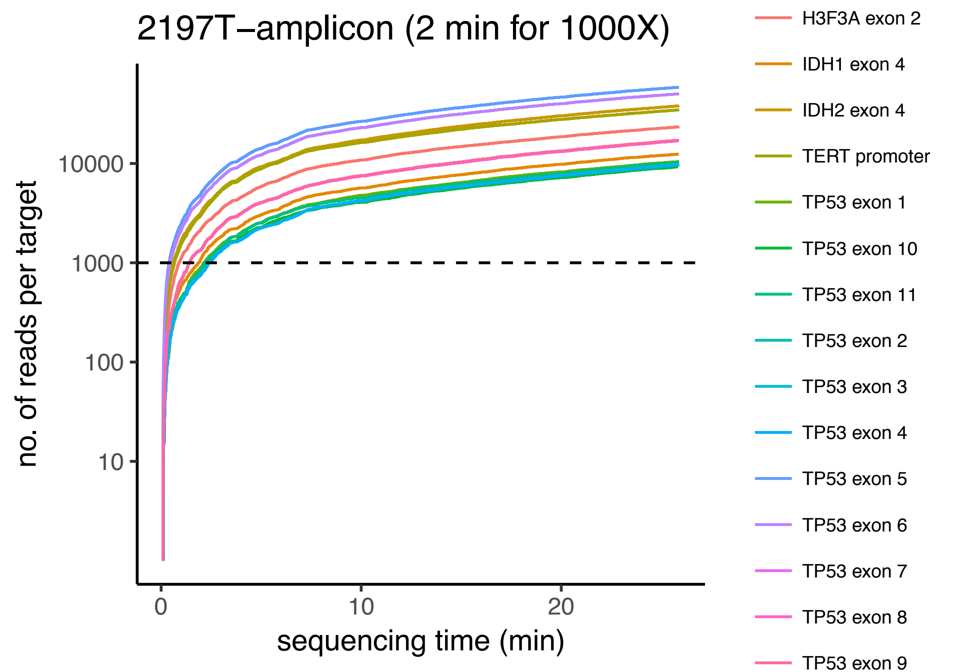
2197T–amplicon (2 min for 1000X)
no. of reads per target
10000
1000
100
10
0
10
20
sequencing time (min)
H3F3A exon 2
IDH1 exon 4
IDH2 exon 4
TERT promoter
TP53 exon 1
TP53 exon 10
TP53 exon 11
TP53 exon 2
TP53 exon 3
TP53 exon 4
TP53 exon 5
TP53 exon 6
TP53 exon 7
TP53 exon 8
TP53 exon 9
